# The ALDH2/PolG2 axis enhances mitochondrial biogenesis via transcriptional regulation of Nrf2 and promotes chemotherapy resistance in acute myeloid leukaemia

**DOI:** 10.1038/s41419-025-07927-z

**Published:** 2025-08-13

**Authors:** Xiuying Hu, Tianzhen Hu, Shuyun Cao, Li Jiang, Yan Zhou, Qin Fang, Jishi Wang

**Affiliations:** 1 Department of Hematology, Affiliated Hospital of Guizhou Medical University, Guizhou, China; 2 School of Basic Medical Sciences, Guizhou Medical University, Guizhou, China; 3 Hematological Institute of Guizhou Province, Guizhou, China; 4 Guizhou Province Hematopoietic Stem Cell Transplantation Centre and Key Laboratory of Hematological Disease Diagnostic and Treatment Centre, Guizhou, China; 5 Department of Pharmacy, Daqin Cancer Hospital of Guizhou Province, Guizhou, China; 6 Department of Pediatric Nephrology and Rheumatology, Affiliated Hospital of Guizhou Medical University, Guizhou, China; 7 Department of Pharmacy, Affiliated Hospital of Guizhou Medical University, Guizhou, China; 8 National Clinical Research Center for Hematologic Diseases, The First Affiliated Hospital of Soochow University, Suzhou, China

**Keywords:** Cancer therapeutic resistance, Transcriptional regulatory elements

## Abstract

Although patients with acute myeloid leukaemia (AML) initially respond to conventional treatments, many patients die from AML progression and relapsed/refractory (RR) disease. Eradicating AML thus remains therapeutically challenging. In this study, we found a strong expression of aldehyde dehydrogenase 2 (ALDH2) and increased mitochondrial biosynthesis in samples from patients with drug-resistant AML, and these changes were strongly associated with poor prognosis and recurrence of AML. We examined the clonogenic capacity, growth and apoptosis of AML cells, as well as mitochondrial DNA expression and reactive oxygen species production. Our results revealed that chemotherapeutic agents triggered the activation of NF-E2-related factor 2 (Nrf2) and promoted high expression of ALDH2, mediating the compensatory activation of mitochondrial respiration and resistance to chemotherapeutic agents in RR AML cells. Nrf2 promoted mitochondrial respiration by activating ALDH2 expression and stabilising the expression of DNA polymerase-gamma2 (PolG2) in mitochondria. Inhibition of the Nrf2-ALDH2/PolG2 pathway reduced AML metabolic fitness and oxidative phosphorylation levels, highlighting the key role of this pathway in promoting cell survival. Nrf2 inhibition reduced the translation of ALDH2, induced a unique mitochondrial stress response and inhibited mitochondrial biosynthesis in AML cells. Importantly, tumours in an in vivo xenograft model were sensitive to combined Nrf2 and ALDH2 inhibition. Given the role of the Nrf2-ALDH2/PolG2 pathway in the progression of AML, inhibition of this pathway may prevent disease relapse/resistance and promote sensitisation to chemotherapy.

## Introduction

Acute myeloid leukaemia (AML) is the most common acute leukaemia in adults, accounting for approximately 80% of cases [1, 2]. Despite advances in supportive treatment, a cytarabine- and anthracycline-based regimen in combination with allogeneic stem cell transplantation remains the preferred treatment option. That treatment approach was reported to result in a 5-year overall survival (OS) rate of 30% for adults [3]. Although some new drugs have been approved for the treatment of AML, the overall survival rate is still unsatisfactory [4]. To improve the long-term survival of patients, new low-toxicity and effective drugs that target the vulnerabilities of AML are needed. Mitochondrial biosynthesis maintains energy production to support cell proliferation and cancer development [5]. Mitochondrial homoeostasis and even microenvironment-induced enhancement of mitochondrial transfer play a role in the survival of AML cells [6, 7]. Despite encouraging results from preclinical studies, the clinical benefits of targeting this molecular mechanism in AML patients have not been demonstrated. Moreover, the degree of cross-talk between proteins involved in mitochondrial biogenesis and tumour cells has not been definitively determined [8].

Mitochondria are membrane-bound organelles that serve as the energy source of eukaryotic cells; unlike other organelles, mitochondria have a unique genome (mitochondrial DNA, mtDNA) that encodes the necessary components of the electron transport chain (ETC) [9]. The peptides encoded by mtDNA are subunits of the enzyme complexes involved in the oxidative phosphorylation system, and mtDNA transcription regulation is closely linked to the cellular metabolic state [10]. However, the mechanism of mtDNA function and mitochondrial metabolism regulation in the context of AML disease progression and drug resistance is not fully understood.

Therapy-resistant AML cells rely on mitochondrial metabolism and are driven by a series of genomic mutations or gene regulation processes that occur in bone marrow [11, 12]. In our previous studies, we found that ALDH2 can protect cells from damage by counteracting oxidative stress [13]. It has been reported that bone marrow stromal cells promote the acquisition of stem cell characteristics and resistance to chemotherapeutic agents in AML cells through the nonclassical TGF-β1/p38/ALDH2 signalling axis [14]. However, the specific molecular mechanism by which ALDH2 induces drug resistance in AML has not been fully elucidated; in particular, the functions of ALDH2 in mitochondrial metabolism and biosynthesis have not been reported. Here, we performed comprehensive bioinformatics analyses of transcriptomic data from 161 AML patients obtained from the TCGA database. Our analysis revealed a significant inverse correlation between ALDH2 expression levels and clinical prognosis in AML patients (*p* < 0.05). Pathway enrichment analysis of differentially expressed genes further revealed strong associations between ALDH2 expression and critical metabolic pathways, particularly those involved in mitochondrial energy metabolism and oxidative phosphorylation. Through comprehensive in vitro and in vivo experiments, we identified ALDH2 as a critical mediator of Ara-C resistance. We found that ALDH2 regulated mitochondrial oxidative phosphorylation, promoted mitochondrial respiration, and thus regulated mitochondrial oxidative stress, and these changes were associated with disease progression. We found that NF-E2-related factor 2 (Nrf2) could increase the expression of ALDH2 and promote mtDNA biosynthesis. mtDNA is located in the mitochondrial matrix and is one of the cellular components most vulnerable to reactive oxygen species (ROS)-mediated damage. The Nrf2/antioxidant response element (Nrf2/ARE) signalling pathway is an important antioxidant stress and cellular protection mechanism [15, 16]. We investigated the molecular mechanism by which Nrf2 promoted high expression of ALDH2 in AML cells under the action of chemotherapy drugs and protected AML cells from damage caused by chemotherapy drugs. These processes jointly promoted the progression of chemotherapy resistance by maintaining mitochondrial biosynthesis and mitochondrial function in AML cells. The heterotrimer Polγ is a DNA replicase that is specific to mitochondria and consists of a single catalytic subunit encoded by DNA polymerase-gamma and a homodimeric helper subunit encoded by the DNA polymerase-gamma2 (PolG2) gene [17, 18]. Here, overexpression of ALDH2 stabilised PolG2 expression in mitochondria and promoted PolG2 localisation to mtDNA. Finally, we found that inhibition of the Nrf2-ALDH2/PolG2 pathway increased the sensitivity of AML cells to Ara-C therapy. Therefore, targeting the Nrf2-ALDH2/PolG2 pathway can both alter mitochondrial respiration and inhibit mtDNA synthesis to disrupt mitochondrial homoeostasis and increase the sensitivity of AML cells to chemotherapy drugs.

## Results

### ALDH2 promotes the proliferation of AML cells and induces drug resistance in a patient-specific manner

**Fig. 1:**
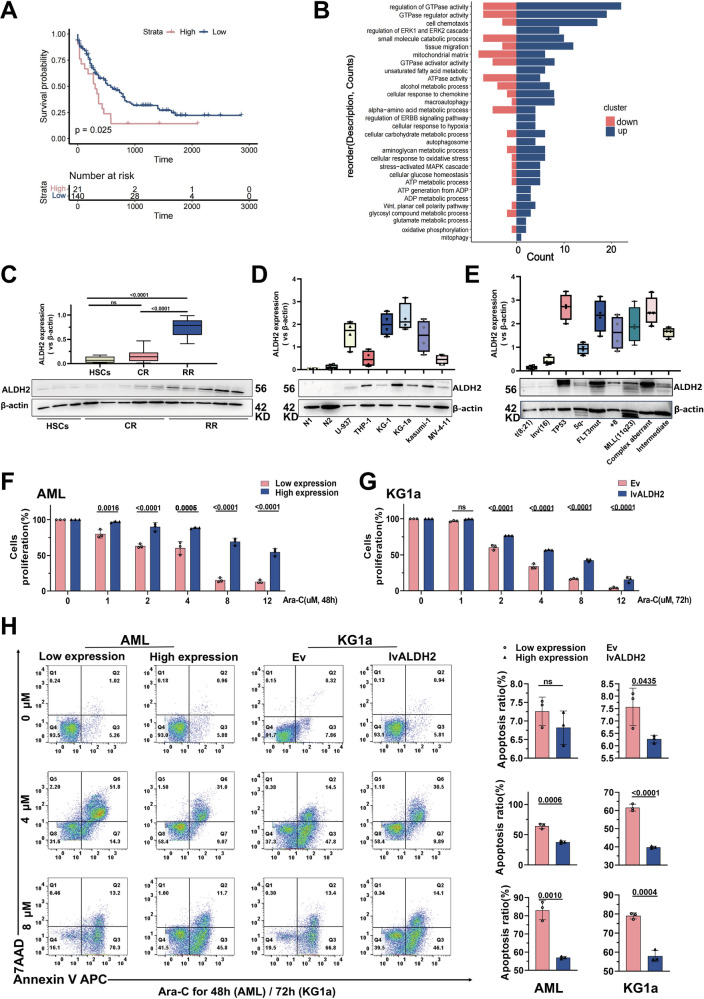
ALDH2 promotes the proliferation of AML cells and induces drug resistance in a patient-specific manner. **A** ALDH2 expression according to the transcriptome sequencing data of 161 AML samples was analysed by bioinformatics analysis. K‒M curves indicating the survival status of patients in the high and low ALDH2 expression groups. **B** The top 30 DEGs in the different ALDH2 high- and low-expression groups were subjected to enrichment analysis of signalling pathways. **C** Representative images showing the changes in ALDH2 expression in the RR (*N* = 20) group and CR (*N* = 20) group after Ara-C treatment. Western blot analysis was used to evaluate the protein levels of ALDH2. The mean value of each protein in the control group was normalised to 1.0, with β-actin serving as the loading control. **D** Representative images showing the changes in ALDH2 expression in normal human cells and different AML cell lines. *n* = 3. **E** Representative images showing the changes in ALDH2 expression in normal individuals and AML patients with different genetic backgrounds. **F** A CCK-8 assay was used to determine the proliferation rate of cells from AML samples from patients with different changes in ALDH2 expression after treatment with Ara-C for 48 h. *n* = 3. **G** The proliferation of KG1a cells was evaluated via a CCK-8 assay after treatment with Ara-C for 72 h. *n* = 3. **H** Flow cytometry was used to explore the apoptosis rates of primary AML cells and KG1a cells in different ALDH2 expression groups after Ara-C treatment for 48 h (AML)/72 h (KG1a). *n* = 3.

The GEO database was searched using the keywords “Acute myeloid leukaemia”, “Microenvironment” and “Resistance”. Drug resistance-related expression profile datasets (GSE152996 and GSE138340) were used for analysis (Fig. S1A). Candidate genes were obtained by fitting the differentially expressed gene groups in the two microarray sets, and the common gene set was screened with the following thresholds: *p* < 0.05 and │log2FC│ > 1.5 (Fig. S1B, D, F). We found that the candidate gene ALDH2 was indeed significantly associated with AML resistance (Fig. S1D). Additionally, the prognostic value of ALDH2 expression for AML patients was analysed using transcriptome and DNA sequencing data from the TCGA database (Fig. S1A, C). For 161 AML patients, there was a significant negative correlation between the ALDH2 expression level and clinical prognosis (*p* < 0.05) (Fig. 1A). After pathway enrichment analysis according to different levels of ALDH2 expression, it was found that ALDH2 was closely related to cellular energy metabolism and mitochondrial metabolic functions; in addition, cell adhesion-, chemokine- and intracellular chemotherapy response-related factors formed a tightly interconnected interaction network (Figs. 1B, S1E). We subsequently selected clinical samples from newly diagnosed AML patients across a variety of morphological (FAB) and molecular subtypes and divided them into chemotherapy-sensitive response (CR, *N* = 20) and relapse-resistant (RR, *N* = 20) groups on the basis of clinical efficacy. ALDH2 expression was significantly upregulated in the RR group (Fig. 1C). In addition, as revealed by the evaluation of AML samples from different cytogenetic backgrounds, AML samples with TP53 mutations, 11q23 rearrangements and complex karyotypes genetic backgrounds associated with poor prognosis presented increased expression of ALDH2 (Fig. 1E). The expression levels of ALDH2 exhibit significant variation across different AML cell lines (Fig. 1D). Among these cell lines, KG1a demonstrates intrinsic resistance to Ara-C (Fig. S1G), while U937 and THP1 show greater sensitivity. Based on these results, we selected KG1a, KG1, and U937 for subsequent gene knockdown experiments, and KG1a, KG1, and THP1 for gene overexpression studies. The effect of Ara-C on AML samples with different ALDH2 expression levels was determined via the CCK-8 method. After treatment with 1, 2, 4, 8, or 12 μM Ara-C for 48 h, the viability of the cells in the groups with low ALDH2 expression decreased by approximately 26.8%, 27.9%, 53.8%, and 41.49%, respectively, compared with those in the groups with high ALDH2 expression (Fig. 1F). To further explore the potential mechanism by which ALDH2 regulates the progression and drug resistant of AML, we tested the sensitivity of AML cell lines transfected with an ALDH2-overexpressing lentivirus to the commonly used chemotherapy drug Ara-C. Compared with empty vector-transfected AML cells, ALDH2-overexpressing lentivirus-transfected KG1a cells presented lower sensitivity to Ara-C (Fig. 1G). AML cells and KG1a cells with high ALDH2 expression all presented smaller changes in the proportion of Annexin V^+^ cells after Ara-C treatment, demonstrating enhanced resistance to Ara-C (Fig. 1H). Conversely, in the cell lines (KG1a, KG1, and U937 cells) with ALDH2 gene downregulation, sensitivity to Ara-C was significantly greater than that observed in cells transfected with the empty vector (Fig. S1H–J). The proportion of Annexin V^+^ cells significantly increased in cells in the ALDH2 gene downregulation groups (Fig. S1K). These results indicate that ALDH2 indeed promotes the drug resistance of AML. Targeting ALDH2 or related pathways may provide an effective strategy for overcoming chemotherapy resistance in AML.

### ALDH2 maintains mtDNA-encoded gene expression and mitochondrial mass

**Fig. 2:**
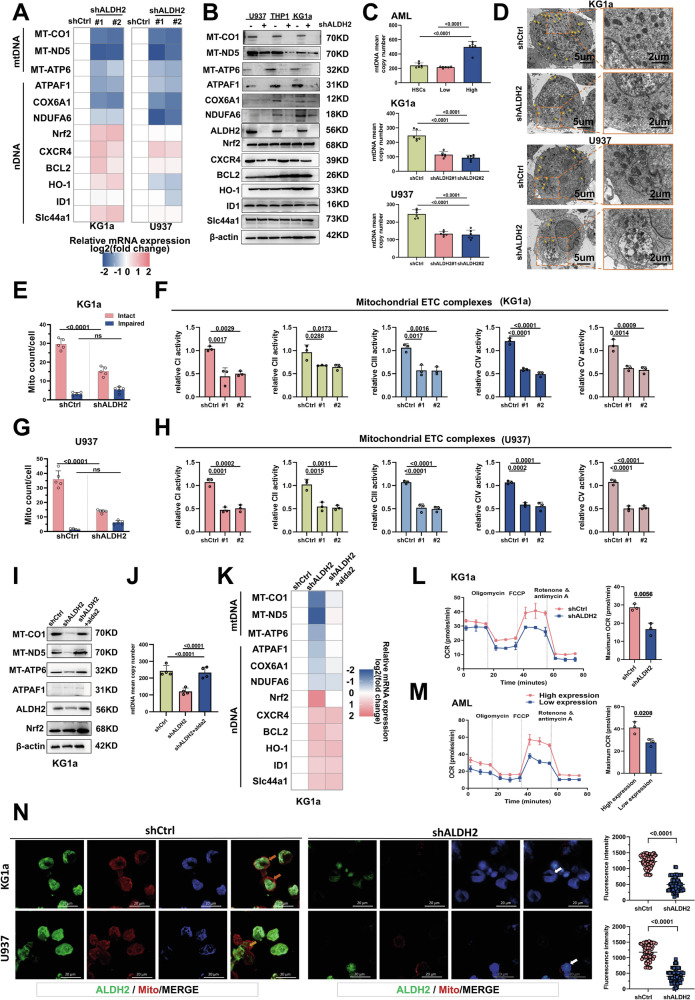
ALDH2 maintains the expression of mtDNA-encoded genes and mitochondrial mass*.* **A** Total RNA was extracted from KG1a and U937 cells with controlled or stable ALDH2 knockdown. The relative mRNA expression of mtDNA- and nDNA-encoded genes was quantified by qPCR and displayed on a log_2_ scale (*n* = 3, mean ± SEM). **B** The expression of proteins encoded by mtDNA and nDNA was detected by Western blotting. *n* = 3. **C** mtDNA abundance in different ALDH2-expressing AML cells and cell lines. *n* = 3. **D**–**H** A representative image (**D**) is shown, with arrows representing mitochondria. Scale bars: 5 μm, 2 μm. Control and ALDH2-knockdown KG1a and U937 cells were analysed by transmission electron microscopy. The mitochondrial quantity and mass (**E**, **G**) were determined. **F**, **H** Mitochondria isolated from control cells or ALDH2-knockdown KG1a and U937 cells were evaluated to determine ETC complex activity. Complex activity was normalised to total mitochondrial proteins. *n* = 3. **I**–**K** Total RNA was extracted from the control group or KG1a cells subjected to stable knockdown of ALDH2, and the relative mRNA expression of mtDNA and nDNA-encoded genes (**I**), the protein expression of mtDNA and nDNA-encoded genes (**J**) and mtDNA abundance (**K**) were detected after treatment with the ALDH2 activator alda2. **L**, **M** The oxygen consumption rates of ALDH2-knockdown cells (**L**) and AML (**M**) cells were determined. **N** The mitochondrial fluorescence intensities of KG1a and U937 cells in the control group and ALDH2-knockdown cells were detected by immunofluorescence staining; *n* ≥ 60 different cells.

Based on previous pathway enrichment analysis, ALDH2 was identified as a key participant in multiple metabolic pathways (Fig. 1B). Since ALDH2 is present mainly in the mitochondrial matrix, we speculated that ALDH2 may modulate mitochondrial function. ALDH2 has been reported to exert a protective effect on DNA by influencing the level of oxidative stress [19]. However, it is not known whether mtDNA plays a role in this process. Mitochondrial metabolic pathways and enzymes are associated with the expression of mtDNA-encoded genes. First, we investigated the specific effect of ALDH2 on mitochondrial biogenesis and further analysed the correlation between the levels of ALDH2 and mtDNA-encoded mitochondrial metabolism-related genes and proteins (Figs. 2A–C, S2A–E). After transfecting AML cell lines with ALDH2-knockdown or ALDH2-overexpressing lentiviruses, we found that the loss of ALDH2 in AML cell lines decreased the mRNA and protein expression of mtDNA-coding genes (Figs. 2A, B, S2A). The mitochondrial respiratory complex enzymes MT-CO1, MT-ND5 and MT-ATP6 are encoded by mtDNA, and the expression of these proteins also changes substantially after the knockdown or overexpression of ALDH2. The levels of nuclear factors and chemokines that control mitochondrial oxidative stress, including Nrf2, HO-1 and CXCR4, did not significantly change. Moreover, we evaluated changes in mtDNA copy number, and after the downregulation of ALDH2 expression, the mtDNA copy number also changed, while after the upregulation of ALDH2, the copy number showed a significant increase. (Figs. 2C, S2B, D, E). These results suggest that ALDH2 maintains the expression of genes encoded by mtDNA. To verify the role of ALDH2 in mitochondrial biogenesis and mitochondrial function, electron microscopy analysis was performed to observe the number and mass of mitochondria in KG1a and U937 cells after ALDH2 knockdown. In ALDH2-knockdown cells, the number of intact mitochondria was reduced by at least 50%, and the number of damaged mitochondria increased nearly threefold (Figs. 2D, E, G, S2F, G). We further isolated mitochondria from stably transfected cell lines and examined the activities of the mitochondrial respiratory chain complex enzymes I-V (Figs. 2F, H, S2J). The downregulation of ALDH2 expression significantly decreased the catalytic activities of these five complexes. mtDNA transcription and mitochondrial protein expression levels were restored after the administration of the ALDH2 activator Alda-1 (Fig. 2I–K). In addition, we verified that the cellular oxygen consumption rate (OCR), which is related to mitochondrial respiration, tended to decrease in the group with low ALDH2 expression (Figs. 2L, M, S2H, I). Immunofluorescence analysis also revealed that the mitochondrial fluorescence intensity was significantly lower in the ALDH2-knockdown group than in the control group (Fig. 2N). Together, these data validate the effects of ALDH2 on mitochondrial biogenesis and mitochondrial function.

### ALDH2 enhances mitochondrial biosynthesis to support leukaemia cell proliferation in vivo

**Fig. 3:**
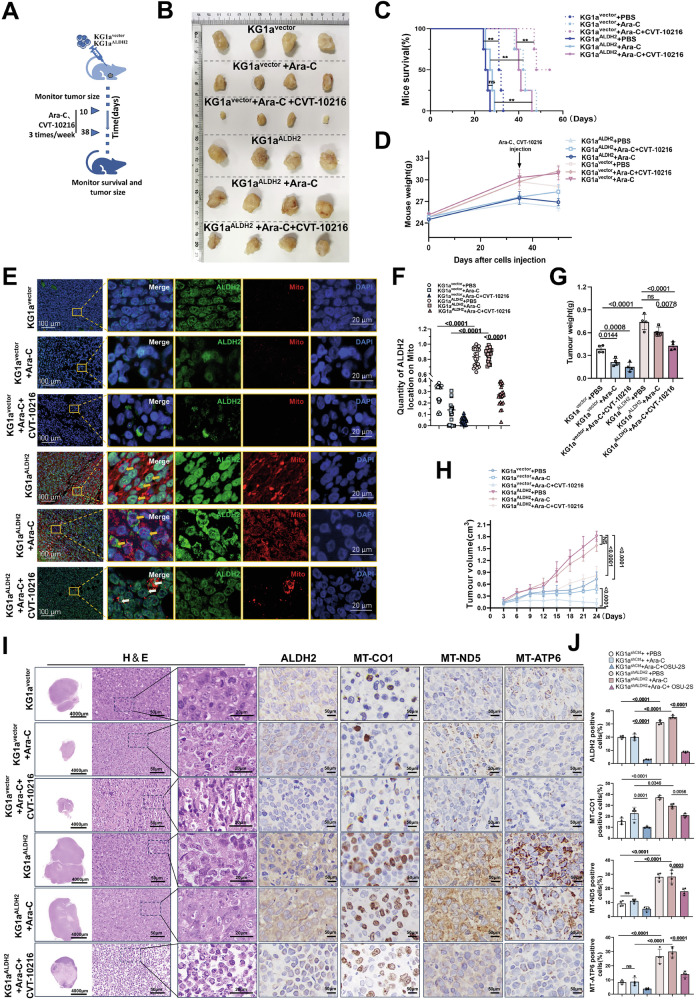
ALDH2 influences mitochondrial biosynthesis to support leukaemia cell proliferation in vivo. **A** Procedures for subcutaneous tumour model establishment in KG1a mice. KG1a cells transfected with the empty vector lentivirus or the ALDH2-overexpression vector were subcutaneously injected into NCG mice (5 × 10^6^ cells per mouse). After 30 days, the cells were given an intraperitoneal injection of 60 mg/kg Ara-C and/or 7.5 mg/kg CVT-10216 or PBS (3 times a week) for an additional 10 days. **B** The appearance of subcutaneous tumour tissue in each mouse group. **C** Overall survival of each treated mouse (*n* = 4 mice per group). *P*-values were calculated with the log-rank test. **: *p* < 0.01. **D** Weights of the mice in each group. **E**, **F** Immunohistochemical staining and histograms of subcutaneous tumour tissue samples showing the coexpression proportions of ALDH2- and Mito-positive cells. **G**, **H** The volume curves (**H**) and weights (**G**) of the subcutaneous tumour tissue in each group are shown. **I**, **J** HE-stained sections (**I**) of the subcutaneous tumour tissue from each mouse group and immunohistochemical staining of ALDH2, MT-CO1, MT-ND5 and MT-ATP6 (**J**) in the subcutaneous tumour tissue sections (each group of 20 images was analysed, *n* = 4 mice per group).

In view of the effect of ALDH2 on mitochondrial biogenesis and mitochondrial respiration, we investigated the effect of ALDH2 on leukaemia proliferation and the underlying mechanism in mice in vivo (Fig. 3A). ALDH2 was overexpressed in KG1a cells (KG1a^ALDH2^), and a mouse subcutaneous tumour model was subsequently established. On the 10th day after subcutaneous tumour transplantation, the control group and ALDH2-overexpression group were administered 0.9% normal saline, Ara-C (60 mg/kg) or Ara-C combined with the ALDH2 inhibitor CVT-10216 (5 μg/kg) three times a week for 4 consecutive weeks, and the tumour growth and physical fitness of the mice were monitored. The results revealed that in the ALDH2-overexpression group, tumour cell proliferation was significantly increased, and the mice exhibited obvious resistance to Ara-C (Fig. 3B). Inhibition of ALDH2 expression restored the ability of Ara-C to inhibit the growth of tumour cells, and the survival and health status of the mice were significantly improved (Fig. 3B–D). The results of immunofluorescence staining of tumour tissue sections indicated that the mitochondrial fluorescence intensity increased significantly in the ALDH2-overexpression group, and the proportion of exhibiting fluorescence also tended to increase (Fig. 3E, F). Monitoring of the tumour volume and weight of the mice revealed that ALDH2 significantly promoted the growth and proliferation of leukaemia cells and significantly increased resistance to Ara-C treatment (Fig. 3G, H). The subcutaneous tumour cells in the ALDH2-overexpression group were intact and compact. Compared with that in the empty vector group, an effect of Ara-C treatment was not obvious in the ALDH2-overexpression group; however, in the CVT-10216 combined with Ara-C group, tumour cell apoptosis was obvious, accompanied by significant tissue vacuolation and cell fragmentation (Fig. 3I). We subsequently investigated the relationship between the expression levels of ALDH2 and the mitochondrial respiratory enzyme-related proteins MT-CO1, MT-ATP6 and MT-ND5 in the animal model. The expression levels of these proteins in the tumour tissues from the KG1a^ALDH2^ group were significantly greater than those in the empty vector group. Moreover, the expression of these genes also decreased with the expression of ALDH2 after ALDH2 expression was inhibited (Fig. 3I, J). In conclusion, these results suggest that ALDH2 upregulation significantly increases resistance to Ara-C and promotes tumour proliferation, accompanied by increased mitochondrial content and higher expression levels of proteins encoded by mtDNA. Inhibiting the expression of ALDH2 also reduced cell proliferation. This finding is consistent with the results of in vitro cell proliferation experiments.

### Nrf2 promotes high expression of ALDH2 and is essential for maintaining mtDNA biosynthesis and respiration by stabilising PolG2 localisation in mitochondria

**Fig. 4:**
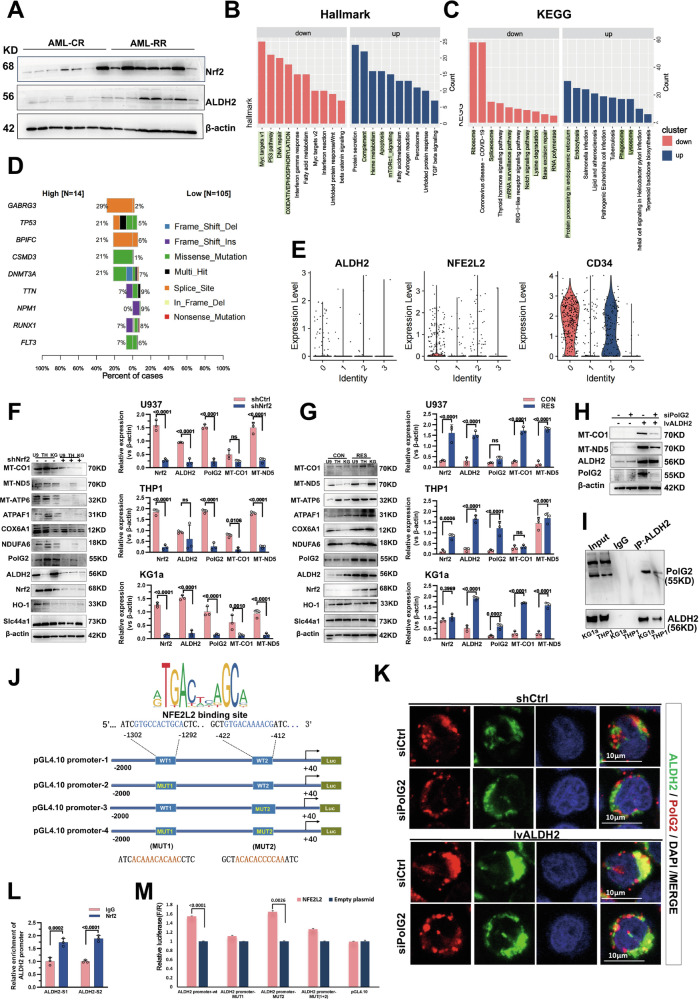
Nrf2 promotes high ALDH2 expression and is essential for maintaining mitochondrial DNA biosynthesis and respiration by stabilising PolG2 localisation to mitochondria. **A** The protein expression levels of ALDH2 in AML cells in different Nrf2 expression groups were examined. *n* = 3. **B** The top 20 pathways associated with different levels of Nrf2 expression were analysed by hallmark enrichment. **C** The top 20 pathways associated with different Nrf2 expression levels were analysed by KEGG enrichment. **D** Analysis of the effects of gene mutations on Nrf2 expression. **E** Subcellular expression of Nrf2 and ALDH2 was analysed by single-cell sequencing. **F**, **G** The expression levels of proteins encoded by mitochondrial DNA and nuclear genes in different cell lines were examined after the regulation of Nrf2 (**F**) and induction of drug resistance (**G**) (*n* = 3). **H** Expression of PolG2 after the regulation of ALDH2. **I** The interaction between ALDH2 and PolG2 in KG1a cells was verified by CoIP. **J** Conservation analysis of DNA binding sequences, possible specific Nrf2 binding targets in the ALDH2 promoter and different luciferase reporter gene expression vectors. **K** The PolG2 fluorescence intensity in KG1a cells subjected to modulation of ALDH2 expression was detected by immunofluorescence staining; *n* ≥ 60 different cells. **L** ChIP was used to detect Nrf2-specific binding sites in the ALDH2 promoter in vivo. Rabbit IgG (negative control) or Nrf2 (positive control) with 10% of the total input chromatin was used as the control. **M** Two wild-type and mutant reporter gene plasmids containing the ALDH2 promoter cloned upstream of luciferase and the reporter vector pGL4.10 were constructed (with Renilla luciferase as an internal reference). The cells were subsequently transfected with the Nrf2 expression vector for 48 h, after which a dual-luciferase reporter gene analysis was performed. The firefly luciferase activity was proportional to the strength of the promoter. The results represent the mean ± standard deviation of 3 separate tests.

AML is a genetically heterogeneous malignancy accompanied by enhanced mitochondrial oxidative phosphorylation (OXPHOS) in therapy-resistant cells, resulting in a dependence on mitochondrial respiration [11, 20]. We previously detected high expression of ALDH2 in AML samples with specific cytogenetic abnormalities, and this phenotype was associated with poor prognosis. In our clinical samples, we also found that in the RR group, the expression level of the nuclear gene Nrf2 (nuclear factor (erythroid-derived 2)-like 2, also known as NFE2L2) correspondingly affected the expression of ALDH2 (Figs. 4A, S3A, B). The expression of both ALDH2 and Nrf2 was highly elevated in the RR group (Fig. S3A, B). Transcriptome data from AML patients in the TCGA were subsequently analysed, and according to pathway enrichment analysis, Nrf2 expression was related to DNA gene repair, mRNA transcription, oxidative phosphorylation and lipid metabolism (Fig. 4B, C). Similarly, high expression of Nrf2 was significantly correlated with P53 and DNMT3A mutations (Fig. 4D). In AML samples, the expression of Nrf2 was generally upregulated, but that of ALDH2 was widespread (Fig. S3C–E). The correlation coefficient between ALDH2 and Nrf2 expressions is relatively high (Fig. S3D). Analysis of the single-cell sequencing dataset GSE126068 revealed that the expression levels of ALDH2 and Nrf2 were greater in CD34^+^ cell populations of the same class (Fig. 4E). Nrf2, a transcription factor that controls the expression of antioxidant response genes and maintains the redox balance of cancer cells, is also activated in various types of cancers [21]. It is associated with low survival and treatment resistance. To determine whether Nrf2 regulates the expression of ALDH2 as a transcription factor, Nrf2 knockdown lentiviruses were transfected into different AML cell lines. A significant reduction in the expression levels of ALDH2 and proteins associated with mitochondrial metabolism was observed in these AML cell lines (Fig. 4F). Moreover, we also observed similar trends in AML cell lines with induced resistance, and these effects were most obvious in KG1a cells (Fig. 4G).

Subsequently, dual-luciferase and ChIP‒PCR assays were performed. Through Jaspar website analysis, we identified two potential Nrf2 binding motifs, which are located upstream of the translation initiation site at −1302 ~ −412 bp (Fig. 4J). The in vivo occupancy of the ALDH2 promoter by Nrf2 in KG1a cells was investigated via ChIP analysis. DNA fragments associated with the transcription factor Nrf2 were obtained via agarose gel electrophoresis of samples from KG1a cells, and the DNA fragments were concentrated in the range of 200–1000 bp, with the strongest enrichment of fragments in the range of 300–500 bp. In addition, the ChIP results revealed that Nrf2 specifically interacted with these binding sites in the ALDH2 promoter region (Fig. 4J, L). The presence of binding sites in the DNA fragments from the ALDH2 promoter region was verified by qPCR. The results demonstrated that in KG1a cells, Nrf2 might bind to two mutation sites in the ALDH2 promoter region to varying degrees. Two distinct truncated ALDH2 promoter fragments and reported mutation sites containing different Nrf2 binding sites were generated from the wild-type (WT) ALDH2 promoter. These fragments were subsequently cloned and inserted into a pGL4-Basic luciferase vector (Fig. 4J). At 48 h post-transfection, the cells were lysed for dual-luciferase reporter gene analysis. According to the luciferase activity value of the NFE2L2 + ALDH2 promoter-WT group, the constructed sequence has promoter activity. Compared with that in the control group, the luciferase activity in the NFE2L2 + ALDH2 promoter-WT group was increased to 156.04%, indicating that the transcription factor NFE2L2 may increase the activity of the ALDH2 promoter (*p* < 0.0001, Fig. 4M). The luciferase activity of the NFE2L2 + ALDH2 promoter-mut1 group returned to 111.81%, and that of the NFE2L2 + ALDH2 promoter-mut1 + 2 group returned to 126.56%. These studies suggest that Nrf2 may promote the expression of ALDH2 by interacting with the Nrf2 binding site (mut 1) in the ALDH2 promoter.

In animal cells, mtDNA is replicated by a DNA polymerase g (Polg) complex consisting of a 140 kDa large catalytic subunit (p140) and a smaller 55 kDa helper subunit (p55). The homodimer PolG2 helper subunit of mitochondrial DNA polymerase γ (Polγ) enhances DNA binding and DNA synthesis by Polg-catalysed subunits. PolG2 also directly binds to DNA and participates in the synthesis of mtDNA [17, 18]. In this study, the expression levels of PolG2, MT-CO1 and MT-ND5 all changed significantly after modulation of ALDH2 expression (Figs. 4H, S3F). Immunofluorescence analysis was performed to evaluate the localisation of PolG2 to mitochondria. PolG2 exhibited strong fluorescence in the mitochondria of the ALDH2-overexpression group, and the fluorescence intensity decreased after PolG2 downregulation (Fig. 4K). Coimmunoprecipitation (CoIP) experiments confirmed the interaction between ALDH2 and PolG2 in the KG1a and THP-1 cell lines (Fig. 4I). Through mitochondrial stress tests, we further verified that in cells with high ALDH2 expression, OCR values also decreased after knockdown of PolG2 (Fig. S3G, H). These results suggest that Nrf2 promotes high expression of ALDH2 and may maintain mtDNA biosynthesis and respiration by stabilising PolG2 localisation to mitochondria.

### Nrf2-ALDH2 regulates mitochondrial metabolism to support leukaemia cell proliferation

**Fig. 5:**
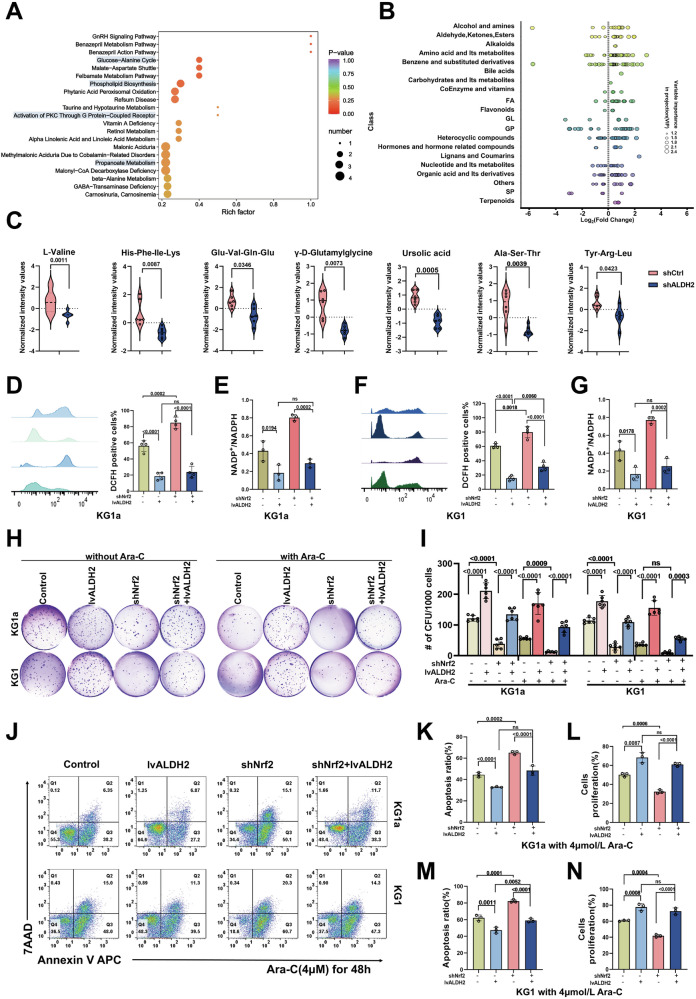
Nrf2-ALDH2 regulates mitochondrial metabolism to support leukaemia cell proliferation. **A** Pathway maps of the top 21 HMDB primary pathways with *p*-values. **B** The greater the absolute value of the horizontal coordinate is, the greater the difference in the content of the substance between the two groups of samples, and the size of the dot represents the variable importance value in the projection. **C** Metabolite differences between the control and shALDH2 groups were calculated via *t*-tests. Metabolites in the affected oxoacid phosphate metabolic pathway are shown; a significance value less than *p* < 0.05 was adopted for enrichment and pathway analysis. *n* = 6. **D**, **F** Changes in ROS levels in KG1a (**D**) and KG1 (**F**) cells after modulation of ALDH2 and Nrf2 expression after 2 μM Ara-C treatment for 24 h. **E**, **G** Changes in REDOX levels in KG1a (**E**) and KG1 (**G**) cells after modulation of ALDH2 and Nrf2 expression after 2 μM Ara-C treatment for 24 h. *n* = 3. **H**, **I** Representative images of colony formation and the fold change in colony numbers of KG1a and KG1 cells. *n* = 6. **J**, **K**, **M** Flow cytometry was used to explore the apoptosis rate of KG1a and KG1 cells with different ALDH2 and Nrf2 expression levels after Ara-C treatment for 48 h. *n* = 3. **L**, **N** A CCK-8 assay was applied to determine the proliferation rate of KG1a and KG1 cells after modulation of ALDH2 and Nrf2 expression plus Ara-C treatment for 48 h. *n* = 3.

Mitochondrial biogenesis affects the expression of mitochondrial metabolism-related enzymes and strongly affects mitochondrial metabolism and cell survival [22, 23]. To determine whether the Nrf2-ALDH2 pathway influences mitochondrial metabolism, metabolomics analysis was performed on KG1a cells. Principal component analysis (PCA) and partial least squares-discriminant analysis (PLS-DA) revealed obvious clustering between the shCtrl and shALDH2 samples (Fig. S4A). In the ALDH2-knockdown group, the *p*-value after FDR adjustment was ≤0.05; 190 metabolites whose abundance significantly differed between groups showed increased abundance, and 72 metabolites showed decreased abundance, as shown in Fig. S4B. The pathways associated with differentially abundant metabolites in the ShALDH2 group were analysed. After ALDH2 expression was knocked down, the production of metabolites such as those involved in the glucose‒alanine cycle, phospholipid biosynthesis, the activation of PKC through G protein–coupled receptors, and the propanoate metabolism pathway were affected (Fig. 5A). The differentially abundant metabolites included alcohols and amines, aldehydes, amino acids and nucleotides (Fig. 5B). Among them, more metabolites involved in amino acid metabolism showed significantly reduced abundance in the shALDH2 group (Fig. 5C).

ALDH2 knockdown led to more extensive metabolic changes in AML cells, especially in terms of mitochondrial amino acid metabolism, and the changes in mitochondrial function also directly or indirectly affected the growth and proliferation of the cells. In KG1a, KG1, and THP-1 cells incubated with 2 μM Ara-C for 24 h, the ROS fluorescence intensity was significantly increased after Nrf2 knockdown, but that increase was significantly smaller after ALDH2 overexpression (Figs. 5D, F, S4G). We also found that the AML redox balance was disrupted after Nrf2 knockdown, and this effect was weaker after ALDH2 overexpression (Figs. 5E, G, S4H). In view of the influence of mitochondrial respiration on the growth and proliferation of leukaemia cells, we also examined the colony formation ability of AML cells after treatment with Nrf2 and ALDH2 (Figs. 5H, I, S4F). After incubation with or without Ara-C, the colony formation ability of the cells decreased after Nrf2 knockdown but significantly recovered after the restoration of ALDH2 expression. Evaluation of the apoptosis, growth and proliferation of the cells also revealed similar trends (Figs. 5J–N, S4C, D). These results suggest that Nrf2 promotes high expression of ALDH2 and increases resistance to AML by promoting mitochondrial metabolic adaptation.

In some disease models, Nrf2 can also participate in the initiation of autophagy [24]. We also examined whether Nrf2-ALDH2 is involved in the activation of mitochondrial autophagy. We found that regardless of the presence or absence of Ara-C, the autophagy level has no close relationship with the activation of the Nrf2-ALDH2 axis (Fig. S4I). Nrf2-ALDH2/PolG2 jointly participate in the observed increase in mitochondrial biosynthesis and protect mitochondrial DNA; however, under the action of Ara-C, DNA damage also increases. We also investigated whether Nrf2-ALDH2 could promote DNA damage repair while promoting biosynthesis (Fig. S4J, K). The relative expression level of γ-H2AX was used as a marker of DNA damage. We added 4 μmol/L Ara-C and observed that under the action of Ara-C, the expression level of γ-H2AX increased, but the upregulation of ALDH2 or downregulation of PolG2 did not seem to have a strong effect on the occurrence of DNA damage (Fig. S4J, K). Therefore, we speculated that the promotion of metabolic adaptation by ALDH2 is closely related to the promotion of mitochondrial biosynthesis and the synthesis of enzymes related to mitochondrial metabolism by Nrf2-ALDH2/PolG2, with a relatively small effect on the repair of damaged mitochondria and DNA. The protective effect of Nrf2-ALDH2/PolG2 on mitochondria may occur during the early stage of cellular stress by promoting mitochondrial biosynthesis to increase the metabolic adaptability of mitochondria, thereby promoting drug resistance.

### Inhibition of the Nrf2-ALDH2 pathway attenuates mitochondrial metabolism in AML cells and inhibits the proliferation of allograft AML cells in vivo

**Fig. 6:**
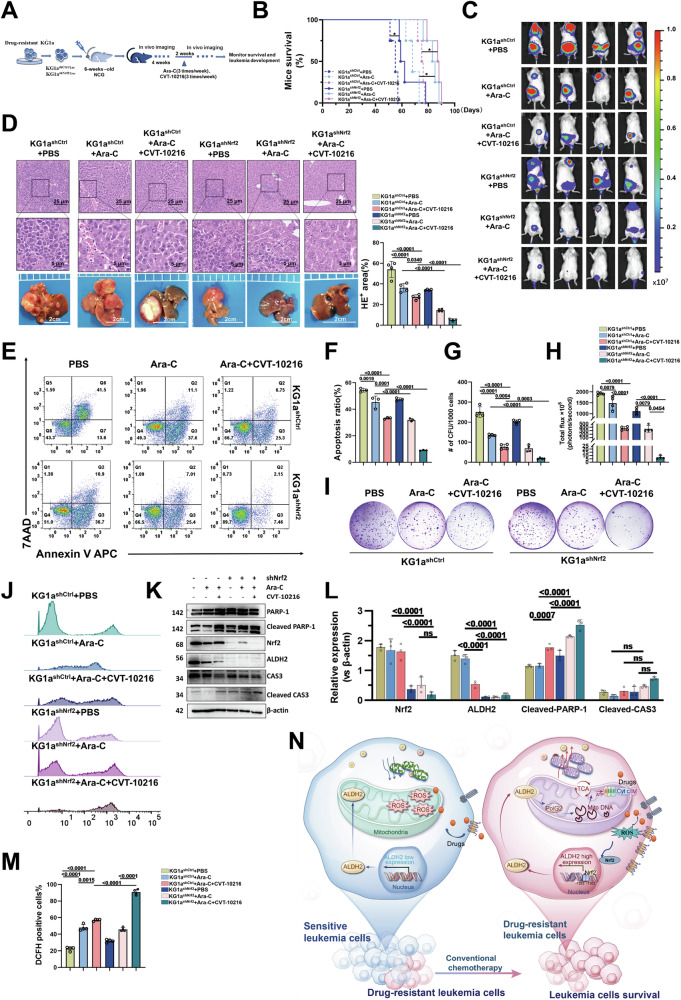
Inhibition of the Nrf2-ALDH2 pathway attenuates mitochondrial metabolism and inhibits the proliferation of allograft AML cells in vivo. **A** Procedures used to construct the AML-CDX mouse model. KG1a cells with high ALDH2 expression were injected into the tail vein of 5–6-week-old NCG mice. **B** Growth curves of the weights of the mice in each group are shown. *P*-values were calculated via the log-rank test. *n* = 4 mice per group. *: *p* < 0.05. **C**, **H** Bioluminescence images of mice taken post-transplantation and intraperitoneal injection treatment and histogram of total flux for each group of mice. **D** The appearance and HE-stained sections of the liver and histograms of the length and weight of the mouse spleen in each group. **E**, **F** Flow cytometry was used to explore the apoptosis rate of tumour cells from mice in different ALDH2 and Nrf2 expression groups after Ara-C treatment. *n* = 3. **G**, **I** Representative images of colony formation and the fold change in colony numbers in tumour cells from mice. *n* = 4. **J**, **M** Changes in ROS levels in tumour cells from mice in different ALDH2 and Nrf2 expression groups after Ara-C treatment. **K**, **L** Detection of apoptosis-related proteins in the tumour cells of mice in different ALDH2 and Nrf2 expression groups after Ara-C treatment. *n* = 3. **N** Working model of Nrf2-mediated induction of high ALDH2 expression, which induces mitosis and mitochondrial remodelling.

We hypothesised that mitochondrial biogenesis and metabolic adaptation induced by the Nrf2-ALDH2 pathway are involved in AML resistance; thus, inhibiting Nrf2-mediated overexpression of ALDH2 may effectively inhibit the growth of AML cells. To this end, we established a humanised leukaemia model by transferring KG1a cells, which were stably transfected with a lentivirus, into sublethally irradiated NCG mice. A low dose of Ara-C and the ALDH2 inhibitor CVT-10216 were administered after 4 weeks (Fig. 6A). In the Nrf2 knockdown group, Nrf2 depletion delayed the development of leukaemia and had a mild inhibitory effect on the proliferation of leukaemia cells (Fig. 6C, D). Greater inhibition was observed in the leukaemia model after combined treatment with CVT-10216 (Fig. 6B–D, H). In addition, this combined treatment significantly increased the survival time of the animals. In the AML xenograft model, combined inhibition of Nrf2 and ALDH2 increased the median survival from 68 days to 87.5 days (a 29% increase in life span). Both in vivo and in vitro data indicated that the Nrf2-ALDH2 pathway was the main pathway involved in promoting leukaemia progression and drug resistance. Nrf2 may not be the single most effective target, and there may be other rescue mechanisms. However, dual-target inhibition of Nrf2 and ALDH2 significantly enhanced the chemotherapeutic effect of low-dose Ara-C on AML. We further observed that after the tumour cells from the mice were extracted, isolated, purified, and cultured in vitro, significantly more cell apoptosis was induced in the group subjected to dual-target inhibition of Nrf2 and ALDH2 combined with Ara-C treatment (Fig. 6E, F). Similarly, as shown in Fig. 6G, I, the combined inhibition of Nrf2 and ALDH2 significantly reduced the colony formation ability of tumour cells in the mice. Cellular ROS levels increased significantly, the mitochondrial oxidation balance was obviously disrupted, and the increase in ROS also increased cell apoptosis (Fig. 6J, M). In this group, the levels of the apoptotic proteins cleaved PARP-1 and cleaved CAS3 also increased significantly (Fig. 6K, L). We then investigated the expression of PolG2 in vivo. The IHC results revealed that the inhibition of Nrf2 and ALDH2 significantly decreased the expression of PolG2 and increased the consumption of PolG2 in mitochondria (Fig. S5A, B). In conclusion, targeting the Nrf2-ALDH2 pathway induces a unique mitochondrial stress response and intensifies cell apoptosis in vivo, and these effects may be initiated after the inhibition of PolG2-induced mitochondrial genesis and mitochondrial respiration.

## Discussion

AML is a haematologic cancer that originates in the bone marrow and is characterised by the expansion of immature myeloid cells, impaired differentiation, and increased proliferative capacity [25, 26]. The introduction of FLT3, IDH1/IDH2 and BCL-2 inhibitors led to the successful development of precision medicine for AML patients. When used as a single drug, each of these inhibitors exhibits moderate antileukaemia activity. They have also exhibited enhanced clinical effects when administered with drugs that exert broader effects by targeting epigenetic and/or other carcinogenic signalling pathways or in combination with conventional cytotoxic drugs [27, 28]. In particular, the combination of venetoclax, a Bcl-2 inhibitor that targets the antiapoptosis mechanism of mitochondria, with Ara-C or 5-azacitidine can improve the survival rate of AML patients, suggesting that mitochondria are relevant therapeutic targets for AML [29–32]. However, the overall cure rate for this disease remains low, and further investigations for precision treatments that target specific vulnerabilities of AML cells are still warranted. Mitochondrial metabolism has recently emerged as a critical dependency in AML, and mitochondrial respiration plays an important role in the maintenance of AML cell growth and chemotherapy resistance. Drug-resistant AML cells have more mitochondria and are highly dependent on mitochondrial respiration [33, 34]. Although there are currently encouraging preclinical studies of agents targeting mitochondrial metabolism, some preclinical drugs that target metabolism have been shown to have limited therapeutic effectiveness [11]. We hope that uncovering specific energy metabolism-related pathways will open new avenues of research and ultimately provide new treatment options for patients with AML.

ALDH2 overexpression is associated with poor prognosis in multiple solid tumours [35, 36]. ALDH2 belongs to the aldehyde dehydrogenase (ALDH) family, is an important participant in the redox reactions of endogenous and exogenous aldehydes and is involved in the regulation of various intracellular signalling pathways [11, 37]. Increasing evidence indicates that ALDHs are highly expressed in the cancer stem cells of solid tumours [38–40]. ALDH2 high expression can promote stem cell properties, proliferation and migration in lung cancer, breast cancer and other cancers; reduce the degree of DNA damage in cancer cells; and mediate the development of drug resistance [41–43]. Disulfiram (DSF) is an aldehyde dehydrogenase inhibitor that can inhibit the expression of ALDH2 and improve the sensitivity of leukaemia stem cells to chemotherapy drugs [44]. However, studies have also shown that the effects of DSF are not closely related to ALDH2 and that DSF can also inhibit the activity of other ALDHs [45]. ALDH2 has been studied in detail in solid tumours, but has been studied less extensively in malignant haematologic tumours; thus, the mechanism of ALDH2 in AML progression, relapse, and drug resistance is still ambiguous. We analysed the prognostic relationship between ALDH2 expression and AML using transcriptome and DNA sequencing data from the TCGA database. In an independent AML cohort, ALDH2 expression was inversely associated with survival in patients with malignant AML. In this study, we found that ALDH2 is an important molecule in AML resistance. ALDH2 was shown to play an important role in cellular metabolism through bioinformatics analysis. Therefore, the role of ALDH2, an aldehyde dehydrogenase localised to mitochondria, in influencing mitochondrial metabolism and even mtDNA synthesis was explored.

Mitochondria have their own unique genetic material, mtDNA, which is responsible for encoding 13 protein subunits involved in the oxidative phosphorylation system, as well as a complete set of transfer and ribosomal RNAs [10]. While more than 99% of the proteins in mitochondria are encoded by nuclear DNA, maintaining the integrity of mitochondrial DNA is essential for mitochondrial function. Synthesis of the mitochondrial respiratory chain complex is completed via mitochondrial biosynthesis, which is a key physiological process for the maintenance of mitochondrial morphology and function [46, 47]. Mitochondrial biosynthesis has been shown to be a major pathway by which tumour cells regulate oxidative phosphorylation during tumorigenesis and progression [48]. The high expression of mitochondrial proteins and the adaptation of mitochondrial metabolism can lead to the emergence of drug resistance in AML [49, 50]. Our findings demonstrate that the upregulation of ALDH2 significantly increased resistance to Ara-C, resulting in greater proliferation potential, which was accompanied by increased mitochondrial content and increased expression of proteins encoded by mtDNA. High expression of ALDH2 increased the expression of these mtDNA-encoded proteins, improved mitochondrial function to a certain extent, protected the translation of mtDNA-encoded proteins during treatment with chemotherapy drugs, and promoted the occurrence of drug resistance to some degree. Through metabolome analysis, we found that during the development of drug resistance, Nrf2-induced high expression of ALDH2 significantly increased the oxidative phosphorylation level while promoting biosynthesis. For AML cells that depend on the oxidative phosphorylation pathway, this represents an effective way to inhibit the growth and proliferation of tumour cells. It is worth noting that this mitochondrial reprogramming mediated by ALDH2 is consistent with the broader metabolic adaptation in drug-resistant AML, in which the dependence on OXPHOS has been established as a marker of drug tolerance. Mechanistically, during treatment with Ara-C, overexpression of ALDH2 can maintain a relatively high level of mtDNA translation, thereby maintaining the energy transmission capacity to meet energy demands, alleviating mitochondrial dysfunction caused by chemotherapy, and promoting a metabolic state conducive to cell survival, which may be the basis of drug resistance.

In this study, the transcription factor Nrf2 bound to the ALDH2 promoter at the 1292 site, promoted high expression of ALDH2 and caused drug resistance in AML cells. Nrf2 is a transcription factor that controls the expression of antioxidant genes and maintains the redox balance of cancer cells. It is also activated in various types of cancer and is associated with low survival rates and treatment resistance [16, 21, 51]. Under chemical stimulation, Nrf2 may act as a key regulator of mitochondrial biosynthesis [15, 22, 52]. The Nrf2/ARE, BRCA1, and p53 signalling pathways are involved in cross-talk during the maintenance of mtDNA integrity [53]. To date, research on the interaction between ALDH2 and Nrf2 has focused mainly on its role in the antioxidant pathway and has focused mostly on diseases other than haematologic malignancies [54–56]. In a model of alcoholic liver injury, Nrf2 was shown to exert antioxidant effects by upregulating the expression of ALDH2 [57]. However, in the context of haematological malignancies, the regulatory relationship between ALDH2 and Nrf2 and their potential biological functions has not yet been reported.

We identified a novel pathway involved in AML resistance, in which Nrf2 promoted high expression of ALDH2 and stable expression of PolG2 in mitochondria. Nrf2-ALDH2-mediated effects on mitochondrial biosynthesis and metabolic homoeostasis were characterised by impaired mitochondrial DNA synthesis following the inhibition of PolG2, which demonstrates the vulnerability of AML cells to chemotherapy drugs. Notably, compared with Nrf2 inhibition alone, dual-targeted inhibition in AML cells was associated with high sensitivity to the cytotoxic effects of chemotherapy drugs. These results highlight the importance of targeting the Nrf2-ALDH2 pathway to sensitise cells to Ara-C. PolG2 is directly responsible for the replication and transcription of mtDNA and promotes the production of mtDNA to complete mitochondrial biosynthesis [17, 18, 58]. The oligomer PolG2 locates crosslinked DNA and targets forked DNA structures similar to the mitochondrial D-ring [17, 59]. Overexpression of ALDH2 increased the expression of PolG2 in mitochondria and promoted drug resistance. The induction of cell death or apoptosis is associated with the inhibition of PolG2 expression and mitochondrial respiration after the inhibition of the Nrf2-ALDH2 pathway. Based on these findings, we propose that the Nrf2-ALDH2 axis may directly or indirectly lead to increased stability of PolG2, increasing mitochondrial function and mitochondrial homoeostasis in the context of oxidative stress induced by chemotherapy drugs. PolG2 is directly responsible for the replication and transcription of mtDNA and promotes the production of mtDNA to complete mitochondrial biosynthesis [17, 18, 58]. The oligomer PolG2 locates crosslinked DNA and targets forked DNA structures similar to the mitochondrial D-ring [17, 59]. Here, overexpression of ALDH2 increased the expression of PolG2 in mitochondria and promoted drug resistance. The induction of cell death or apoptosis is associated with the inhibition of PolG2 expression and mitochondrial respiration after the inhibition of the Nrf2-ALDH2 pathway. Based on these findings, we propose that the Nrf2-ALDH2 axis may directly or indirectly lead to increased stability of PolG2, increasing mitochondrial function and mitochondrial homoeostasis in the context of oxidative stress induced by chemotherapy drugs.

Ara-C treatment also induces significant DNA damage. The Nrf2/ARE signalling pathway is an important cellular protection mechanism. Nrf2 can regulate the expression of components of the antioxidant system in mitochondria and indirectly protect mtDNA from damage. The effect of Nrf2 on mtDNA repair has been reported to be mediated mainly by base excision repair, which is considered the main repair pathway for the mitochondrial genome [15]. Turnover of the mitochondrial pool due to coordinated processes of mitochondrial biogenesis and mitophagy is an important process in maintaining mitochondrial stability. Nrf2 can also participate in the initiation of autophagy [24, 60]. We investigated whether the Nrf2-ALDH2 axis is involved in the processes of mitochondrial autophagy and DNA damage repair. Interestingly, the Nrf2-ALDH2 axis seems to have little relationship with the regulation of mitochondrial autophagy, and the expression level of γ-H2AX is also not strongly related to the activation of the Nrf2-ALDH2 axis. These results suggest that the mitochondrial protective effects mediated by the Nrf2-ALDH2/PolG2 axis may occur primarily during early cellular stress responses, functioning mainly through enhanced mitochondrial biogenesis to improve metabolic adaptability rather than through postdamage repair mechanisms.

In conclusion, the Nrf2-ALDH2 axis plays important roles in drug resistance and disease progression in AML because of its effect on mitochondrial synthesis. Targeting the Nrf2-ALDH2 pathway induces a unique mitochondrial stress response in AML cells, which may be initiated after the inhibition of PolG2-mediated mitochondrial biosynthesis and mitochondrial respiration, as shown in Fig. 6N. We believe that during the progression of AML, metabolic adaptation of mitochondria induces the resistance of AML cells to chemotherapy drugs. Our data on the targeting of metabolic adaptation-dependent mitochondrial biosynthesis and the activation of specific metabolic pathways provide experimental evidence for the clinical development of new drug targets. Our findings suggest that dual-target Nrf2/ALDH2 inhibition may be well tolerated and be associated with minimal myelotoxicity, significantly enhancing sensitivity to chemotherapy drugs, providing a valuable potential target for the development of effective clinical drugs for AML treatment in the future.

## Materials and methods

### Primary AML samples, cell lines and reagents

Primary AML samples were collected from 40 clinically diagnosed AML patients (see Supplementary Table 1 for details). Among the included patients, 20 patients were in the RR group, and 20 patients were in the remission group. The samples were purified by standard Ficoll density gradient centrifugation before culture. Normal peripheral blood mononuclear cells were obtained from healthy donors (*n* = 12). CD34^+^ cells were sorted by flow cytometry (FACSMelody, BD), with an activity >90% and a purity of 95%. Lentiviral vectors for ALDH2 and Nrf2 knockdown or overexpression were transfected into THP-1, U937 (Guizhou Haematopoietic Stem Cell Transplantation Center Laboratory, China), KG1a (ATCC) and KG1 (Guangzhou Cellcook Biotech Co., Ltd.) cell lines. Drug-resistant cell lines were incubated with high-dose Ara-C for a long period of time. Ara-C (HY-13605), Alda-1 (HY-18936) and CVT-10216 (HY-19801) antibodies were purchased from MedChemExpress Co., Ltd. ALDH2, Nrf2, MT-CO1, MT-ND5, MT-ATP6, ATPAF1, COX6A1, NDUFA6, CXCR4, BCL2, HO-1, ID1, Slc44a1, PolG2, PARP-1, cleaved PARP-1, CAS3, cleaved CAS3, γ-H2AX, LC3b and β-actin antibodies were purchased from AFFINIGY, Immunoway or ProteinTech (See Supplementary Table 3 for details).

### Stable transfection of cell lines and primary AML cells

ALDH2-overexpressing lentiviruses (LV-ALDH2 (46179-1): Ev/lvALDH2), Nrf2-overexpressing lentiviruses (LV-NFE2L2(14619-1)), three ALDH2-knockdown lentiviruses (LV-ALDH2-RNAi (18198-1a/b/c): shCtrl/shALDH2) and a Nrf2-knockdown lentivirus (NFE2L2-RNAi(48516-1)), provided by GENE Corporation (Shanghai, China), and siPolG2(1370-S), provided by HyCyte (Suzhou, China), were transfected into cell lines and primary AML cells according to the manufacturer’s protocol. Before transfection, THP-1 (MOI = 40), U937 (MOI = 40), KG1a (MOI = 200), and KG1 (MOI = 160) cells were seeded in 12-well plates at a density of 5 × 10^4^ cells per well. Polybrene (8 μg/mL) was used to increase the infection efficiency. After 12 h, the medium was replaced with fresh medium. At 72 h, GFP reporter gene expression (transfection efficiency > 90%) was observed via fluorescence microscopy, and the gene manipulation effect was verified by qPCR/Western blotting (overexpression efficiency > 3-fold, knockdown efficiency < 50%).

### Analysis of cell viability and apoptosis

The cell counting kit-8 (CCK-8) method was used to evaluate cell viability and proliferation. Transfected cells were seeded at a density of 1 × 10^4^ cells/well in 96-well plates. After treatment with cytarabine (Ara-C) at concentrations ranging from 0 to 12 μM for 24, 48 or 72 h, 10 μL of CCK-8 (TargetMol, C0005) reagent was added to each well. The cells were incubated at 37 °C for 60–90 min. Absorbance was measured at 450 nm using an enzyme microplate reader (Epoch, Biotek). Apoptotic cells were analysed via Annexin V-FITC/7-AAD staining (AP105, Multisciences, Hangzhou). A total of 1 × 10^6^ cells were collected and washed with PBS, and then 5 μL of Annexin V-FITC and 10 μL of 7-AAD were added for 15 min in the dark. Detection was performed using a flow cytometer (BD FACSCalibur, USA). The threshold was set to exclude debris (FSC/SSC), and the FITC+/7-AAD- phenotype was defined as early apoptosis, whereas the FITC+/7-AAD+ phenotype was defined as late apoptosis. Colony formation experiments were conducted in alkaline methylcellulose medium (MethoCult H4100). A total of 3000 transfected cells were mixed with methylcellulose containing 10% FBS and seeded in 6-well plates. The plates were cultured at 37 °C in a 5% CO_2_ environment for 21 days. Cultures with more than 50 cells were counted, and the colony formation rate was calculated. Each experiment had three replicates, and the experiments were repeated three independent times.

### Real-time polymerase chain reaction (RT‒PCR)

Total RNA was isolated from cells using TRIzol reagent (Invitrogen, Carlsbad, CA, USA). RNA reverse transcription was performed using Fastking gDNA Dispelling RT SuperMix (Tian Gen Biotech, China). A Talent qPCR PreMix (SYBR Green) (Tian Gen Biotech, China) kit was used for RT‒PCR to detect changes in gene expression. The primer sequences are shown in Table S2. mRNA expression was normalised to that of β-actin, and the transcripts were quantified. Fold changes were calculated using the comparative Ct method and expressed as the average of three independent assays.

### Western blot analysis

Proteins in the whole-cell lysates were subjected to sodium dodecyl sulfate‒polyacrylamide gel electrophoresis and transferred to polyvinylidene fluoride membranes. Later, the membranes were incubated with the corresponding primary and secondary antibodies (see Supplementary Table 3 for the antibody dilutions) and visualised with an infrared imaging system (Tanon5200, Shanghai).

### CoIP analysis

The immune complex A protein was precipitated with the rProtein A/G magnetic IP/CoIP kit (AM001-02, ACE Biotechnology, Nanjing). The B protein was detected by Western blotting according to the manufacturer’s instructions. A total of 1000 μg of total protein and 5 μg of anti-ALDH2 antibody (or IgG control) were added, and the mixture was rotated and incubated at 4 °C overnight. Then, 20 μL of protein A/G magnetic beads were added, and the mixture was incubated at 4 °C for 2 h. After magnetic separation, the samples were washed with PBS three times and eluted to collect the bound protein. Western blotting was used to detect PolG2 (anti-PolG2, 1:1000; anti-ALDH2, 1:2000) in the precipitate. The experiment was repeated three times.

### Mitochondrial stress evaluation via Seahorse XF analysis

The OCR was measured using a Seahorse XFe12 flux analyser (Agilent Technologies, California, USA). Briefly, 24 h before detection, different groups of drug-treated cells (8000 cells/well) were added to an 8-well hippocampal XFp cell culture plate coated with poly D-lysine. The cell culture plates were placed in a CO_2_-free cell incubator and cultured at 37 °C for 25–30 min. According to the Agilent Seahorse XFp User manual, oligomycin, FCCP, and rotenone/antimycin A were added to measure the OCR and normalise it to the appropriate protein content. Three replicates were included for each condition.

### Mitochondrial isolation and detection of mitochondrial respiratory enzyme activity

A mitochondrial extraction kit (Solarbio, Beijing) was used to extract mitochondria according to the manufacturer’s instructions. Then, functional assays were performed, and proteins were extracted for the detection of mitochondrial respiratory enzyme activity. Mitochondrial respiratory complexes I-V were evaluated with a mitochondrial complex I-V test kit (complex I-BC0515, II-BC3230, III-BC3240, IV-BC0945, V-BC1445; Solarbio, Beijing). Generally, complex I can catalyse the dehydrogenation of NADH to NAD+, and the oxidation rate of NADH at 340 nm was determined to calculate the activity of this enzyme. Coenzyme Q, the catalytic product of complex II, can further reduce 2,6-dichloroindoxol. 2,6-Dichloroindoxol has a characteristic absorption peak at 605 nm. The enzyme activity was calculated by measuring the reduction rate of 2,6-dichloroindoxol. Mitochondrial complex III is also known as COQ-cytochrome C reductase. Unlike oxidised cytochrome C, reduced cytochrome C exhibits characteristic light absorption at 550 nm, so an increased rate of light absorption at 550 nm can be used as an indicator of the activity of the mitochondrial complex III enzyme. Mitochondrial complex IV, also known as cytochrome C oxidase, catalyses the conversion of reduced cytochrome C to oxidised cytochrome C, so a decrease in the rate of light absorption at 550 nm can be used as an indicator of the activity of the mitochondrial complex IV enzyme. Mitochondrial complex V, also referred to as F1F0-ATP synthase, hydrolyses ATP to produce ADP and Pi. The activity of complex V was determined by measuring the rate of increase in Pi levels. Absorbance values were measured with an automatic microplate reader (Epoch, Biotek), and three independent experiments were performed for each detection.

### Liquid chromatography‒tandem mass spectrometry (LC–MS) nontargeted metabolome analysis

Metabolomic analysis was supported by Jingjie PTM BioLabs (Hangzhou, China). The samples were repeatedly freeze-thawed on ice and liquid nitrogen to extract metabolites, and the cell metabolite concentrations were quantitatively determined using an LC‒MS/MS-based metabolomics platform (Orbitrap Exploris™480, Thermo Fisher Scientific). The XCMS program was used for peak extraction, peak sequence correction and retention time correction. Differences in metabolite levels between the control and treatment groups were determined by one-way analysis of variance (ANOVA), with an FDR < 0.05 suggesting statistical significance; moreover, pathway enrichment and functional analyses were performed for the metabolites with significant differences.

### Measurement of NADP+/NADPH

The NADP+/NADPH redox ratio was determined using an enhanced NADP+/NADPH assay kit (S0180S, Beyotime, Shanghai), which is a colour development reaction based on WST-8, according to the manufacturer’s protocol. The amounts and ratio of NADP+ (oxidised Coenzyme II) and NADPH (reduced Coenzyme II) in cells, tissues and other samples were measured by colorimetry. Before testing, each sample was divided into two parts. One part was used to measure the total amounts of NADP+ and NADPH, and the other part was treated at 60 °C for 30 min to remove NADP+. The samples were treated with GAPDH at 37 °C for 10 min and incubated with WST-8 at 37 °C for an additional 30 min. Absorption was detected at a wavelength of 450 nm.

### Cellular ROS assay

Intracellular ROS levels were detected using the fluorescent probe DCFH-DA (S0033M; Beyotime Biotechnology). After treatment, the number of cells in each group was measured by flow cytometry (FACSLyric, BD, USA) according to the manufacturer’s instructions. The treated cells from each group (1 × 10⁶ cells/mL) were collected and washed twice with PBS; then, 10 μM DCFH-DA was added, and the mixture was incubated at 37 °C in the dark for 30 min. After washing with PBS to remove free probe, the cells were resuspended, and the fluorescence intensity (excitation wavelength, 488 nm; emission wavelength, 525 nm) was immediately detected with a flow cytometer (BD FACSLyric). A negative control and positive control were used. Each experiment was independently repeated 3 times.

### Morphological analyses of mitochondria

The cells were fixed with 2% glutaraldehyde, and the number and microstructure of mitochondria were subsequently evaluated via transmission electron microscopy (TEM) (HITACHI HT7800, Hitachi, Japan). ImageJ was used to quantitatively analyse the number and morphology of the mitochondria.

### Immunofluorescence analyses

Cell lines stably transfected with Nrf2 and/or ALDH2 knockdown or overexpression lentiviruses were incubated with the corresponding antibodies and subjected to fluorescence staining, and confocal immunofluorescence images were captured using an LSM800 confocal laser scanning microscope (ZEISS, Germany).

### Quantitative analysis of the mtDNA copy number

The Human Mitochondrial DNA (mtDNA) Monitoring Primer Set (7246, Takara) was used to analyse mtDNA via RT‒PCR. The mtDNA copy number was measured on the basis of nuclear DNA (nDNA) and quantified according to the difference in Ct values between mtDNA and nDNA.

### Chromatin immunoprecipitation and quantitative PCR (ChIP‒qPCR)

The cells were fixed with 1% paraformaldehyde at room temperature for 10 min, and the reaction was terminated with glycine. After the cells were washed with PBS (containing protease inhibitors), they were lysed in cell lysis buffer on ice for 15 min and then centrifuged to obtain the cell nuclei. The chromatin was sonicated to obtain 200–1000 bp fragments. After the fragment sizes were verified via electrophoresis, the chromatin was incubated with an anti-Nrf2 antibody (ab62352, Abcam, 4 μg) at 4 °C overnight and then combined with protein A/G magnetic beads for 2 h. The complexes were washed successively with low-salt, high-salt, LiCl and TE buffers. The protein‒DNA complexes were eluted, the cross-links were denatured at 62 °C for 2 h, and the complexes were inactivated at 95 °C. After the DNA was purified, qPCR was used to detect the promoter region of the target gene (the primer sequences are shown in Table S3), and the fold enrichment was calculated using the %Input method. An IgG negative control was used for each group, with 3 technical replicates for each group, and the experiment was repeated independently 3 times to ensure reproducibility.

### Dual-luciferase reporter gene assay

Wild-type and mutant reporter plasmids containing the ALDH2 promoter were constructed with the pGL3 basic promoter. Then, (1) the NFE2L2/Control plasmid (pCDNA3.1) +ALDH2 promoter-wt +pRL-tk; (2) the NFE2L2/Control plasmid +ALDH2 promoter-MUT1 +pRL-tk; (3) the NFE2L2/Control plasmid +ALDH2 promoter-MUT2 +pRL-tk; (4) the NFE2L2/Control plasmid +ALDH2 promoter-MUT (1 + 2) +pRL-tk; and (5) the NFE2L2/Control plasmid +pGL4.10+pRL-tk were transfected into HEK293T cells inoculated into 96-well plates at 2 × 10^4^ cells/well. Later, 20 μL of the transfection complex mixture [mixed A (50 ng of overexpression plasmid + 100 ng of reporter plasmid + pRL-TK 20 ng+MEM 10 μL) and B (MEM 10 μL + liposome 2000 0.2 μL)] was added to each well. The luciferase activities of the samples were determined using the Promega Dual Luciferase Reporting Assay System (E1910).

### Xenotransplantation experiment in mice

Female NOD/ShiLtJGpt-*Prkdc*^em26Cd52^*Il2rg*^em26Cd22^/Gpt severe immunodeficiency (NCG) mice were obtained from China Jiangsu GemPharmatech Co., Ltd. All animal experiments were conducted in accordance with the agency’s animal welfare guidelines.

To study the effects of ALDH2 on the growth, proliferation and drug resistance of leukaemia cells in vivo, a mouse subcutaneous tumour model was established. In brief, KG1a^vector/Luc^ and KG1a^ALDH2/Luc^ cells (5 × 10^6^/mouse) were first mixed with Matrigel (3432-005-01, R&D Systems Bio-Techne) at a 1:1 ratio. The mixture was subsequently injected subcutaneously into the left axilla of 5–6-week-old NCG mice. Ten days after transplantation, the mice were intraperitoneally injected with 0.9% normal saline, Ara-C (60 mg/kg), or Ara-C combined with the ALDH2 inhibitor CVT-10216 (7.5 mg/kg) three times a week for 2 weeks. During this period, subcutaneous tumour volume and survival were monitored.

To investigate the role of the Nrf2-ALDH2 axis in chemotherapy resistance, a mouse whole-body infiltration CDX model was established for in vivo analysis. Specifically, 5–6-week-old immunodeficient NCG mice were injected with KG1a ^shCtrl/Luc^ or KG1a ^shNrf2/Luc^ cells (5 × 10^6^/mouse) via the tail vein. After successful modelling, the mice with leukaemia were treated with the ALDH2 inhibitor CVT-10216 (5 mg/kg) alone or in combination with low-dose Ara-C (30 mg/kg), and those in the control group were treated with normal saline. In vivo mouse imaging (IVIS) was conducted to monitor the leukaemia load and observe the survival time and health status of the mice. The number of AML cells in the bone marrow of the mice in each group was determined by flow cytometry.

### Haematoxylin & eosin (HE) and immunohistochemical (IHC) staining

For the subcutaneous tumour formation experiments, the subcutaneous tumours of the mice in each group were collected. Thereafter, liver, spleen, kidney and femur samples from CDX mice were collected, fixed, paraffin-embedded, and then stained with HE. Changes in cell morphology and staining were subsequently observed under an optical microscope (NIKON ECLIPSE E100, Japan). Sections for IHC analysis were dewaxed, followed by antigen retrieval, blocking and incubation with specific antibodies. Finally, the sections were photographed under an optical microscope.

### Bioinformatics analysis of RNA/DNA-seq data from public datasets

The expression profiles of ALDH2 and Nrf2 in normal and AML samples were acquired from the TCGA and Genotype-Tissue Expression Project (GTEx) databases. The RNA and DNA sequencing (RNA/DNA-seq) data, overall survival rates and bioinformatics data were downloaded from public datasets (AML RNA/DNA-seq data from TCGA-LAML and normal RNA/DNA-seq dataset from the GTEx database). The type and frequency of Nrf2 gene mutations in AML, the survival of AML patients and the correlation between ALDH2 and Nrf2 expression were analysed, and the differentially expressed genes were subjected to Gene Ontology (GO) and Kyoto Encyclopedia of Genes and Genomes (KEGG) analyses.

### Statistical analysis

Statistical analysis was performed using GraphPad Prism 9 (GraphPad, La Jolla, CA, USA). All values are expressed as the mean and standard deviation (SD). Differences between the two groups were evaluated by two-way ANOVA or Student’s *t*-test. *p* < 0.05 was considered to indicate statistical significance.

## Supplementary information

Supplementary document Legends

Table S1. AML patient sample information

Table S2. Primer sequence information

Table S3. Antibody information

Fig. S1 High expression of ALDH2 promotes the proliferation of AML cells and induces drug resistance in a patient-specific manner.

Fig. S2 ALDH2 maintains mtDNA-encoded gene expression and mitochondrial mass.

Fig. S3 Nrf2 promotes high ALDH2 expression and is essential for maintaining mitochondrial DNA biosynthesis and respiration by stabilising PolG2 localisation to mitochondria.

Fig. S4 Nrf2-ALDH2 regulates mitochondrial metabolism to support leukaemia cell proliferation.

Fig. S5 Inhibition of the Nrf2-ALDH2 pathway attenuates the mitochondrial metabolism and inhibits the proliferation of allograft AML cells in vivo.

Western blot original data

## Data Availability

All data generated and analysed during this study are included in this published article and are available on request.
