# Supplementary material for: The ALDH2/PolG2 axis enhances mitochondrial biogenesis via transcriptional regulation of Nrf2 and promotes chemotherapy resistance in acute myeloid leukaemia
Source: Cell Death Dis. 2025 Aug 13;16(1):616. doi: 10.1038/s41419-025-07927-z (PMC12344002; doi:10.1038/s41419-025-07927-z)
Supplement: Supplementary file 1 — Supplementary document Legends [file 41419_2025_7927_MOESM1_ESM.docx]

**Legends：**

**Table S1. AML patient sample information**

**Table S2. Primer sequence information**

**Table S3.** **Antibody information**

**Fig. S1** **High expression of ALDH2 promotes the proliferation of AML cells and induces** **drug resistance in a patient-specific manner.** **A** Common candidate genes were screened by fitting data from the TCGA database and GEO database, and the bioinformatics analysis process for drug resistance gene identification. **B** The ESTIMATE algorithm estimates the immune matrix correlation score in the microenvironment. Common candidate genes were screened by fitting to the scored immune matrix. **C** The DEGs with FDR ≤ 0.05% between the high and low ALDH2 expression groups were hierarchically clustered, and the change amplitude was 1. **D** Common candidate genes were screened by fitting data from the GEO database. **E** Microenvironment-related differential gene interaction network. **F** The DEGs with FDR ≤ 0.05% between high or low expression of ALDH2 groups showed hierarchical clustering, and the change amplitude was 1 time. **G** A CCK-8 assay was used to determine the proliferation rate of AML cell lines treated with Ara-C for 48 h. n = 3. **H- J** The proliferation of AML cell lines was detected via a CCK-8 assay after ALDH2 knockdown. n = 3. **K** Flow cytometry was used to explore the apoptosis rate of AML cell lines after ALDH2 knockdown combined with different Ara-C treatments for 24 h. n = 3.

**Fig. S2 ALDH2 maintains mtDNA-encoded gene expression and mitochondrial mass*.*** **A** Total RNA was extracted from KG1 cells with controlled or stable ALDH2 knockdown. The relative mRNA expression of mtDNA- and nDNA-encoded genes was quantified by qPCR and displayed on a log_2_ scale. **B**, **D**, **E** Detection of mtDNA abundance in different AML cell lines after the regulation of ALDH2. n = 3. **C** Total RNA was extracted from THP-1 with stable high expression of the ALDH2 gene. The relative mRNA expression of the mtDNA- and nDNA-encoded genes was quantified by qPCR, and the results are presented as the log_2_ values (n = 3, mean ± standard error). **D** The relative mRNA expression of mtDNA- and nDNA-encoded genes was quantified by qPCR, and the results are presented in log2 form (n = 3, mean ± standard error) in THP-1 cells with upregulated ALDH2 gene expression. **F**, **G** The mitochondria in control cells and ALDH2 gene-knockdown KG1 cells were analysed by transmission electron microscopy. Representative images (**F**); arrows indicate mitochondria. Scale bars: 5 μm, 2 μm. The quantity and quality of mitochondria in the six groups were determined (**G**). **H**, **I** The oxygen consumption rate of ALDH2 gene-knockdown KG1 cells was determined. **J** Mitochondria were isolated from control cells or ALDH2 gene-knockdown KG1 cells, and the activity of the ETC complexes was measured. The activity of the complexes was normalized to that of total mitochondrial protein. The data are presented as the means ± standard deviations; n = 3.

**Fig. S3 Nrf2 promotes high ALDH2 expression and is essential for maintaining mitochondrial DNA biosynthesis and respiration by stabilizing PolG2 localization to mitochondria.** **A** ALDH2 expression levels in different Nrf2 expression groups (N = 7 per group). **B** ALDH2 expression (N = 6 per group). **C** The correlations of biological characteristics, cytogenetics, disease progression and other factors with Nrf2 expression in 161 AML patients were analysed. **D** Coexpression of ALDH2 and Nrf2 in various tissues from AML patients. **E** Expression of ALDH2 and Nrf2 in normal subjects and AML patients. **F** For the histogram in Fig. 4 H, it showed relative expression of PolG2 after modulation of ALDH2 expression. **G-J** The oxygen consumption rates in KG1a and AML cells with different expression levels of ALDH2 and PolG2 were determined.

**Fig. S4** **Nrf2-ALDH2 regulates mitochondrial metabolism to support leukaemia cell proliferation.** **A** Unsupervised principal component analysis (PCA) of widely targeted small metabolites. **B** Hierarchical clustering of differentially abundant metabolites in shCtrl- and shALDH2-transfected cells with an FDR of 0.05% and a >2-fold change. **C** A CCK-8 assay was used to determine the proliferation rate of THP1 cells after ALDH2 and Nrf2 regulation with Ara-C treatment for 48 h. n = 3. **D** Flow cytometry was used to explore the apoptosis rate of THP1 cells in different ALDH2 and Nrf2 expression groups after Ara-C treatment. n = 3. **E** The relative contents of the top 50 differentially abundant metabolites are shown. The relative content of differentially abundant metabolites in different samples was standardized by calculating the Z value. **F** Representative images of colony formation and the fold change in colony numbers of THP1 cells. n = 6. **G** Changes in ROS levels in THP1 cells after ALDH2 and Nrf2 regulation. **H** Changes in REDOX levels in THP1 cells after ALDH2 and Nrf2 regulation. n = 3. **I** The protein expression levels of γ-H2AX in KG1a cells were examined in different ALDH2 expression groups after Ara-C treatment for 24 h. n = 3. **J**, **K** The protein expression levels of LC3II and PolG2 in KG1a cells were examined in different ALDH2 expression groups after Ara-C treatment for 24 h. n = 3.

**Fig. S5 Inhibition of the Nrf2-ALDH2 pathway attenuates the mitochondrial metabolism and inhibits the proliferation of allograft AML cells *in vivo*.** **A**, **B** Immunohistochemical staining and histograms of subcutaneous tumour tissue samples showing positive correlations for ALDH2, Nrf2 and PolG2.
