# Supplementary figures and images for: The ALDH2/PolG2 axis enhances mitochondrial biogenesis via transcriptional regulation of Nrf2 and promotes chemotherapy resistance in acute myeloid leukaemia
Source: Cell Death Dis. 2025 Aug 13;16(1):616. doi: 10.1038/s41419-025-07927-z (PMC12344002; doi:10.1038/s41419-025-07927-z)

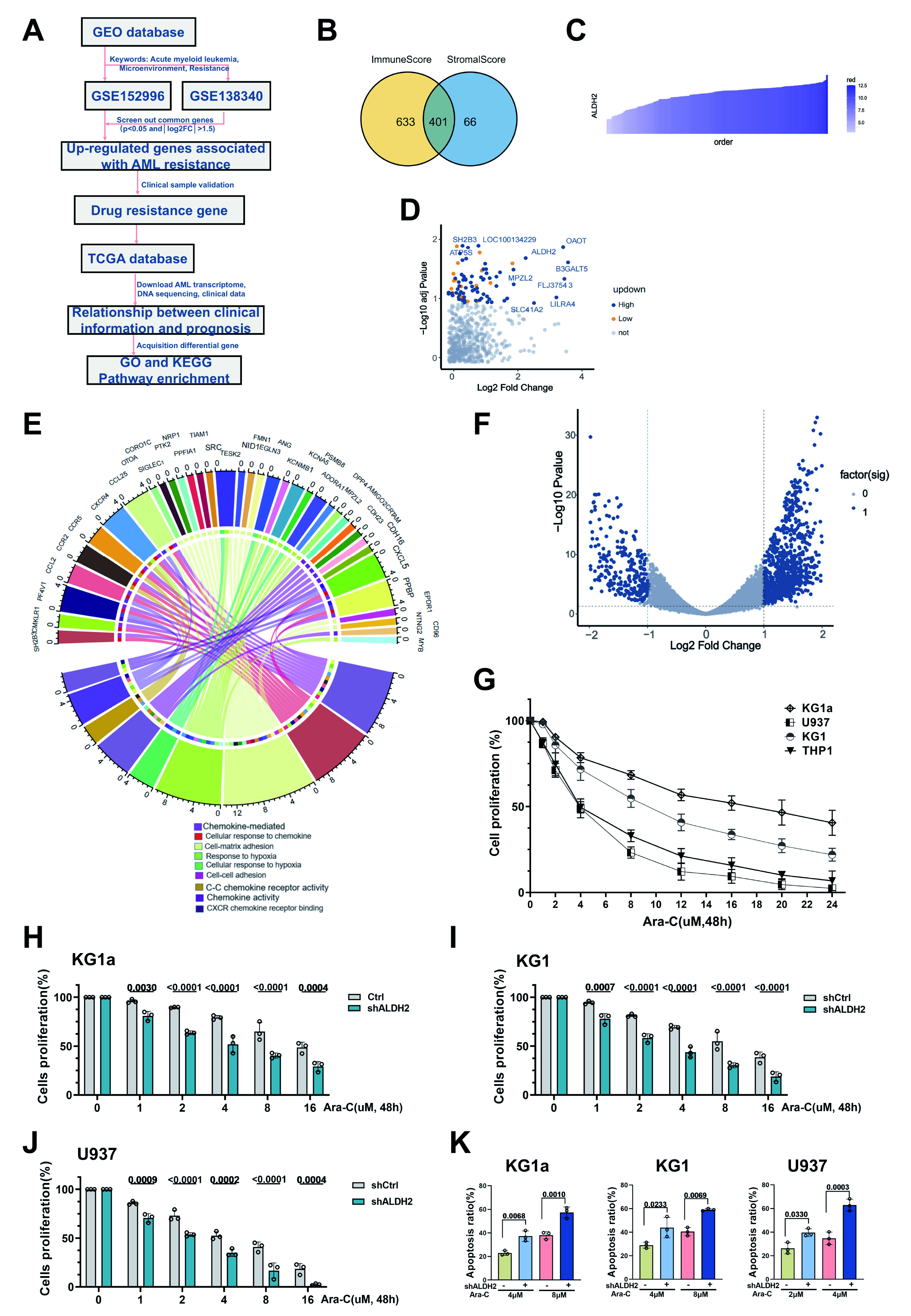

Supplement: Supplementary file 5 — Fig. S1 High expression of ALDH2 promotes the proliferation of AML cells and induces drug resistance in a patient-specific manner. [file 41419_2025_7927_MOESM5_ESM.tif]

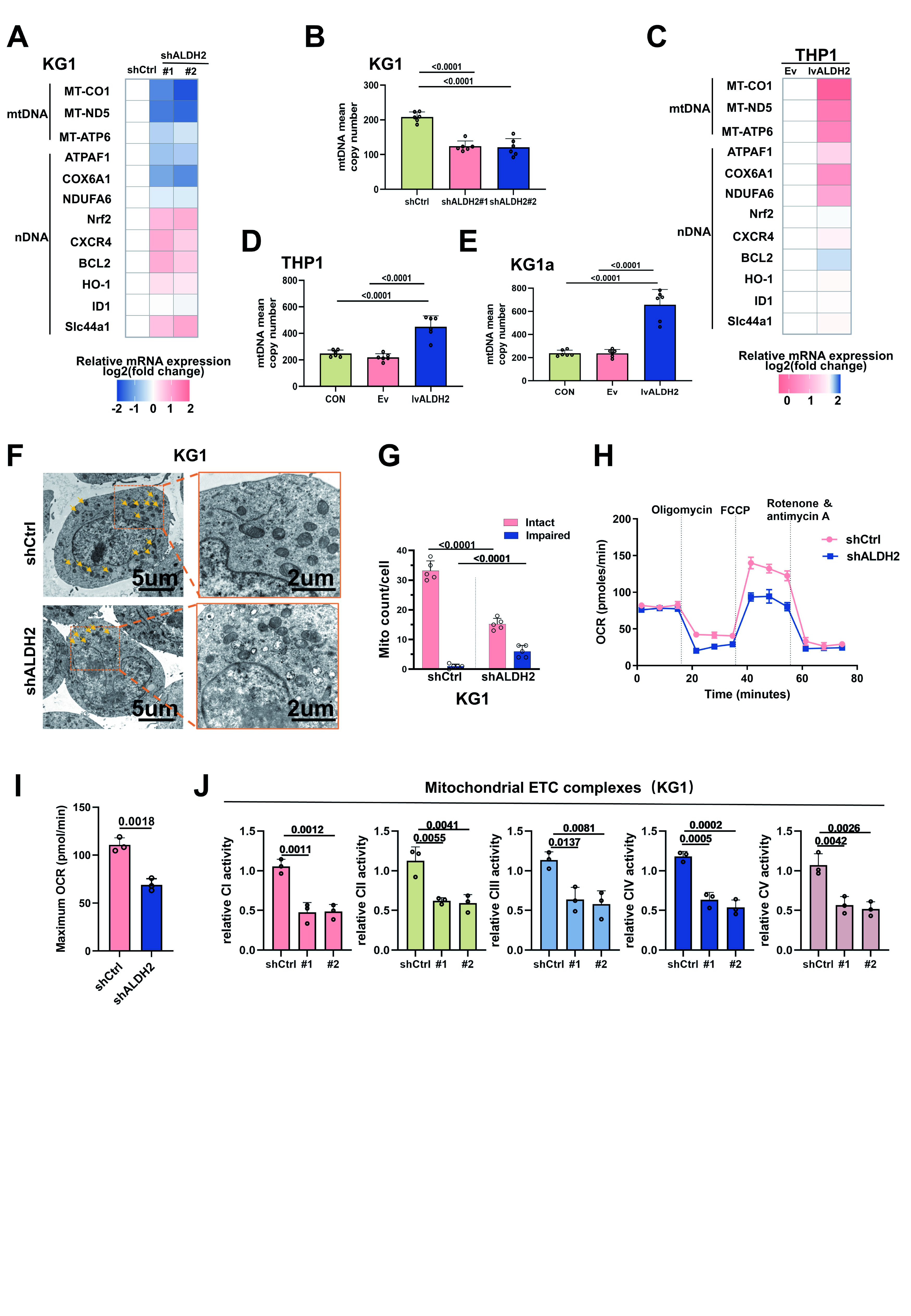

Supplement: Supplementary file 6 — Fig. S2 ALDH2 maintains mtDNA-encoded gene expression and mitochondrial mass. [file 41419_2025_7927_MOESM6_ESM.tif]

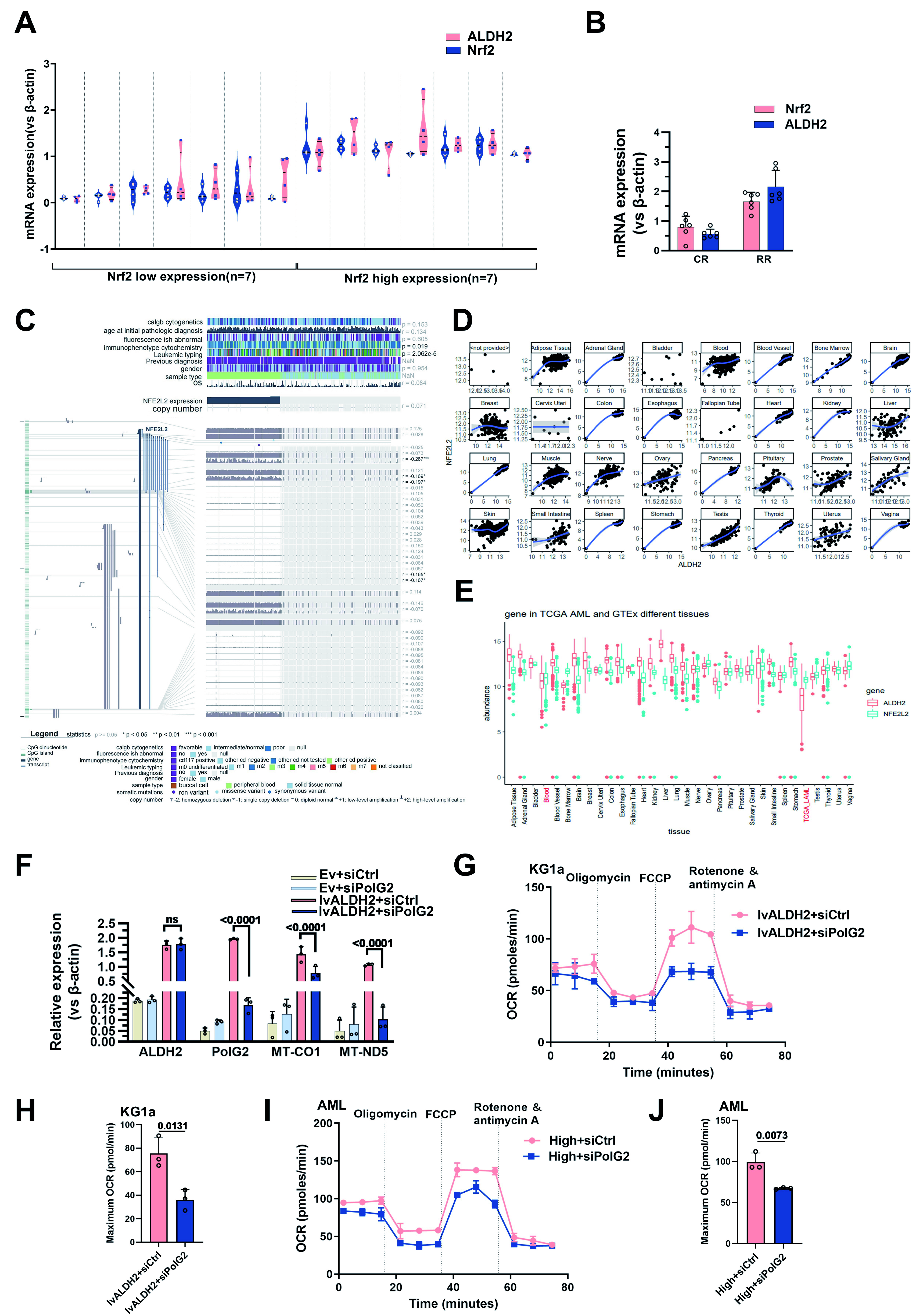

Supplement: Supplementary file 7 — Fig. S3 Nrf2 promotes high ALDH2 expression and is essential for maintaining mitochondrial DNA biosynthesis and respiration by stabilising PolG2 localisation to mitochondria. [file 41419_2025_7927_MOESM7_ESM.tif]

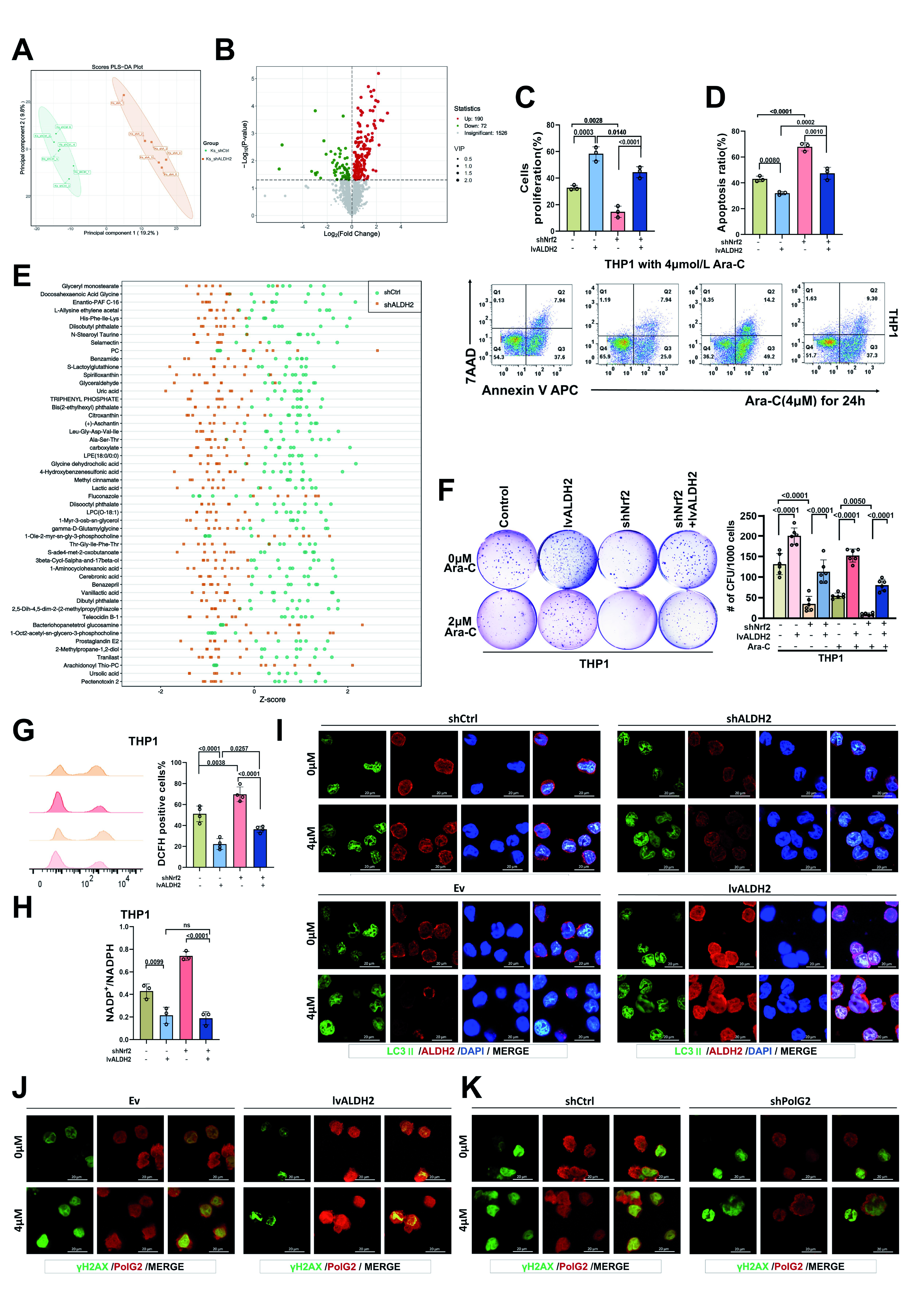

Supplement: Supplementary file 8 — Fig. S4 Nrf2-ALDH2 regulates mitochondrial metabolism to support leukaemia cell proliferation. [file 41419_2025_7927_MOESM8_ESM.tif]

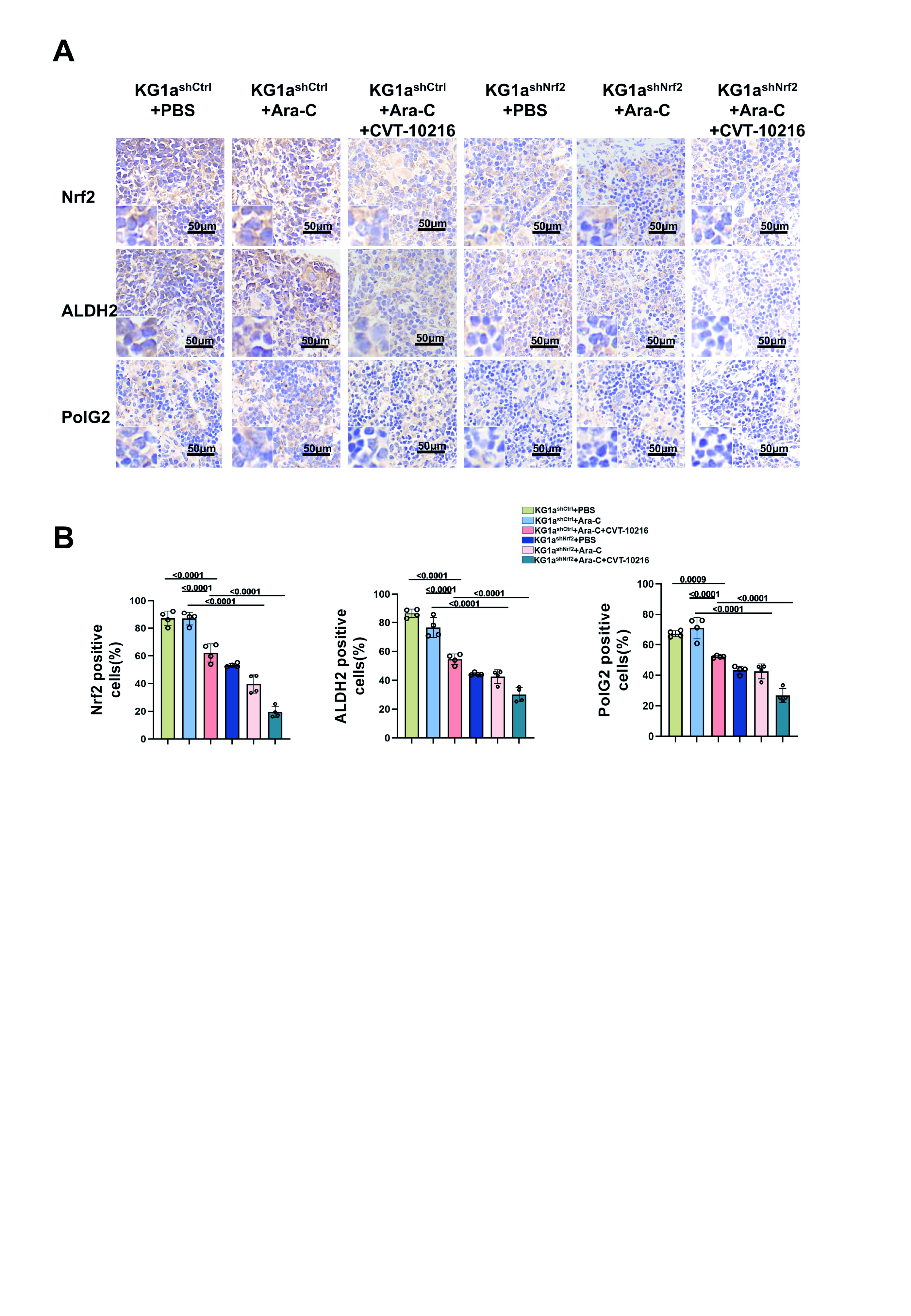

Supplement: Supplementary file 9 — Fig. S5 Inhibition of the Nrf2-ALDH2 pathway attenuates the mitochondrial metabolism and inhibits the proliferation of allograft AML cells in vivo. [file 41419_2025_7927_MOESM9_ESM.tif]
