# Supplementary material for: The ALDH2/PolG2 axis enhances mitochondrial biogenesis via transcriptional regulation of Nrf2 and promotes chemotherapy resistance in acute myeloid leukaemia
Source: Cell Death Dis. 2025 Aug 13;16(1):616. doi: 10.1038/s41419-025-07927-z (PMC12344002; doi:10.1038/s41419-025-07927-z)
Supplement: Supplementary file 10 — Western blot original data [file 41419_2025_7927_MOESM10_ESM.pdf]

Figure 1C

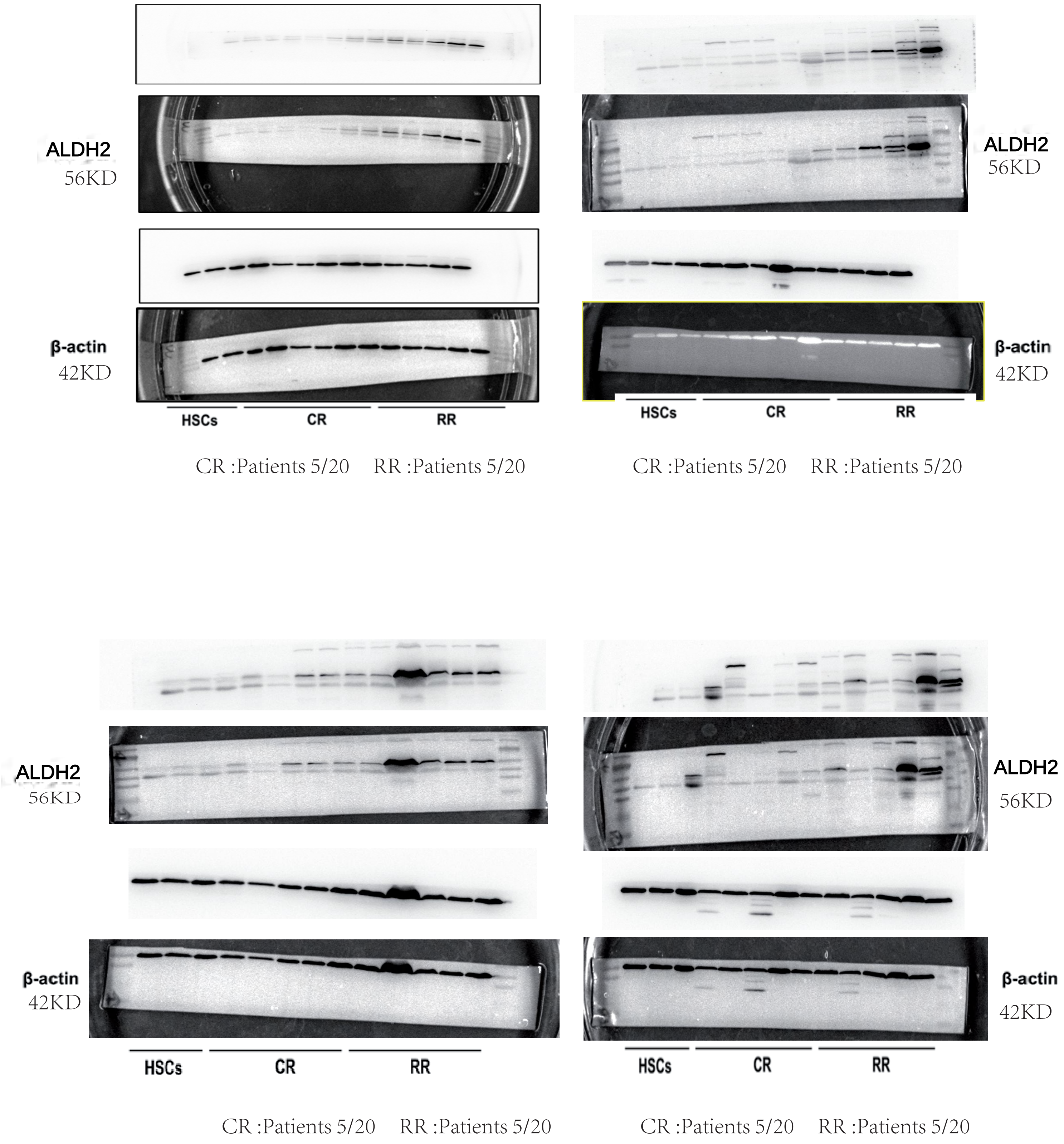

ALDH2
56KD
β-actin
42KD
HSCs
CR
RR
CR :Patients 5/20
RR :Patients 5/20
ALDH2
56KD
β-actin
42KD
HSCs
CR
RR
CR :Patients 5/20
RR :Patients 5/20
ALDH2
56KD
β-actin
42KD
HSCs
CR
RR
CR :Patients 5/20
RR :Patients 5/20
ALDH2
56KD
β-actin
42KD
HSCs
CR
RR
CR :Patients 5/20
RR :Patients 5/20

Figure 1D

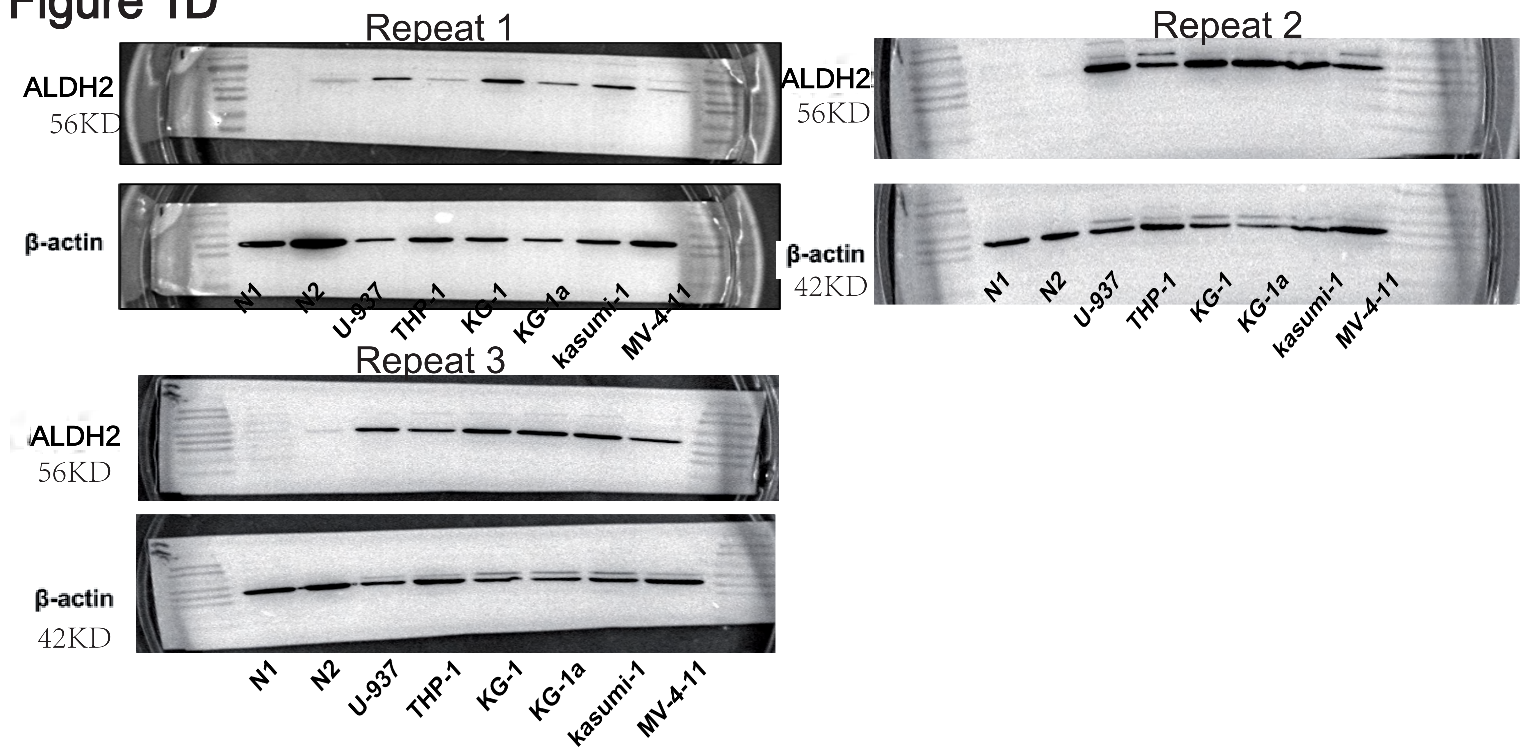

Figure 1E

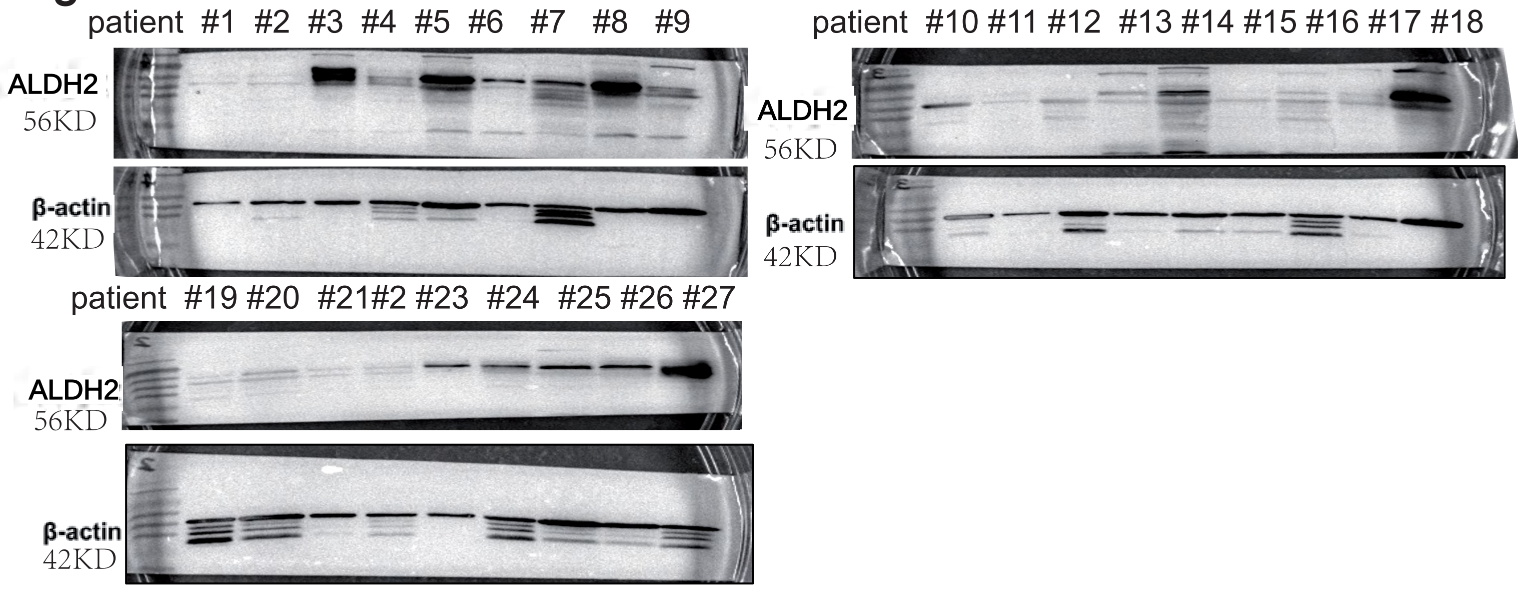

Figure 2B

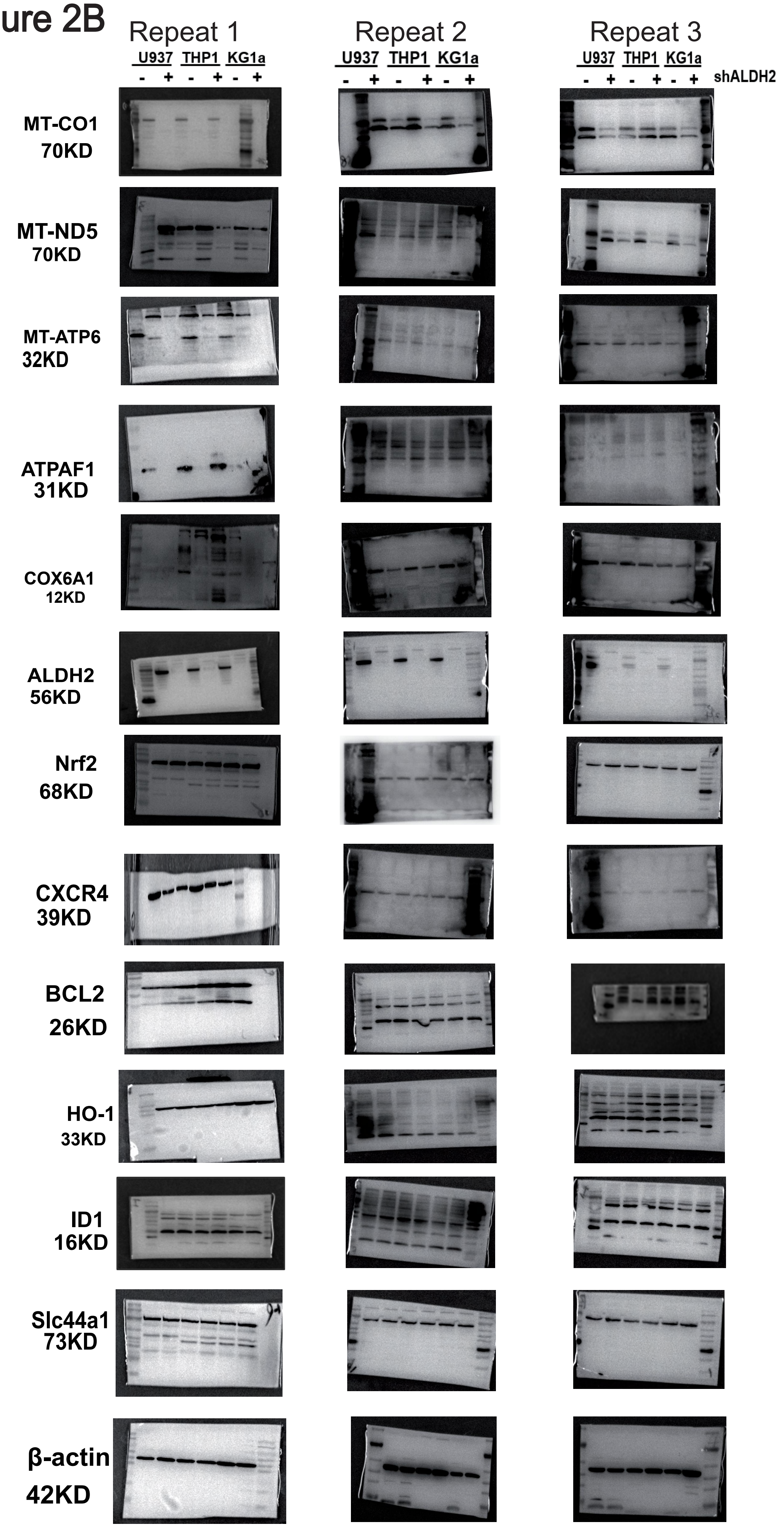
Repeat 1
Repeat 2
Repeat 3
U937 THP1 KG1a
- + - + - +
shALDH2
MT-CO1
70KD
MT-ND5
70KD
MT-ATP6
32KD
ATPAF1
31KD
COX6A1
12KD
ALDH2
56KD
Nrf2
68KD
CXCR4
39KD
BCL2
26KD
HO-1
33KD
ID1
16KD
Slc44a1
73KD
β-actin
42KD

Figure 4A

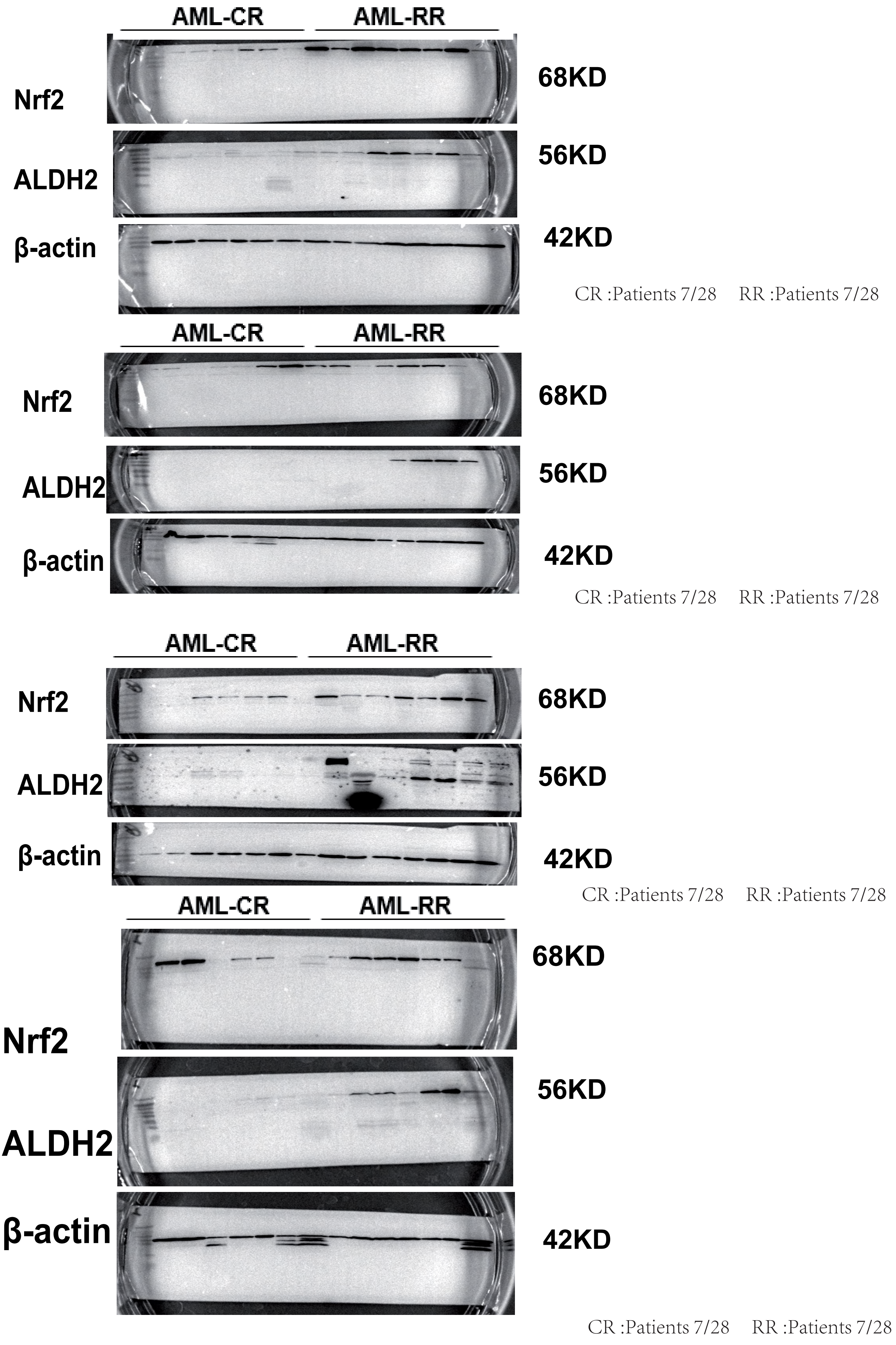
AML-CR
AML-RR
Nrf2
68KD
ALDH2
56KD
β-actin
42KD
CR :Patients 7/28
RR :Patients 7/28
AML-CR
AML-RR
Nrf2
68KD
ALDH2
56KD
β-actin
42KD
CR :Patients 7/28
RR :Patients 7/28
AML-CR
AML-RR
Nrf2
68KD
ALDH2
56KD
β-actin
42KD
CR :Patients 7/28
RR :Patients 7/28
AML-CR
AML-RR
68KD
Nrf2
56KD
ALDH2
β-actin
42KD
CR :Patients 7/28
RR :Patients 7/28

Figure 4F

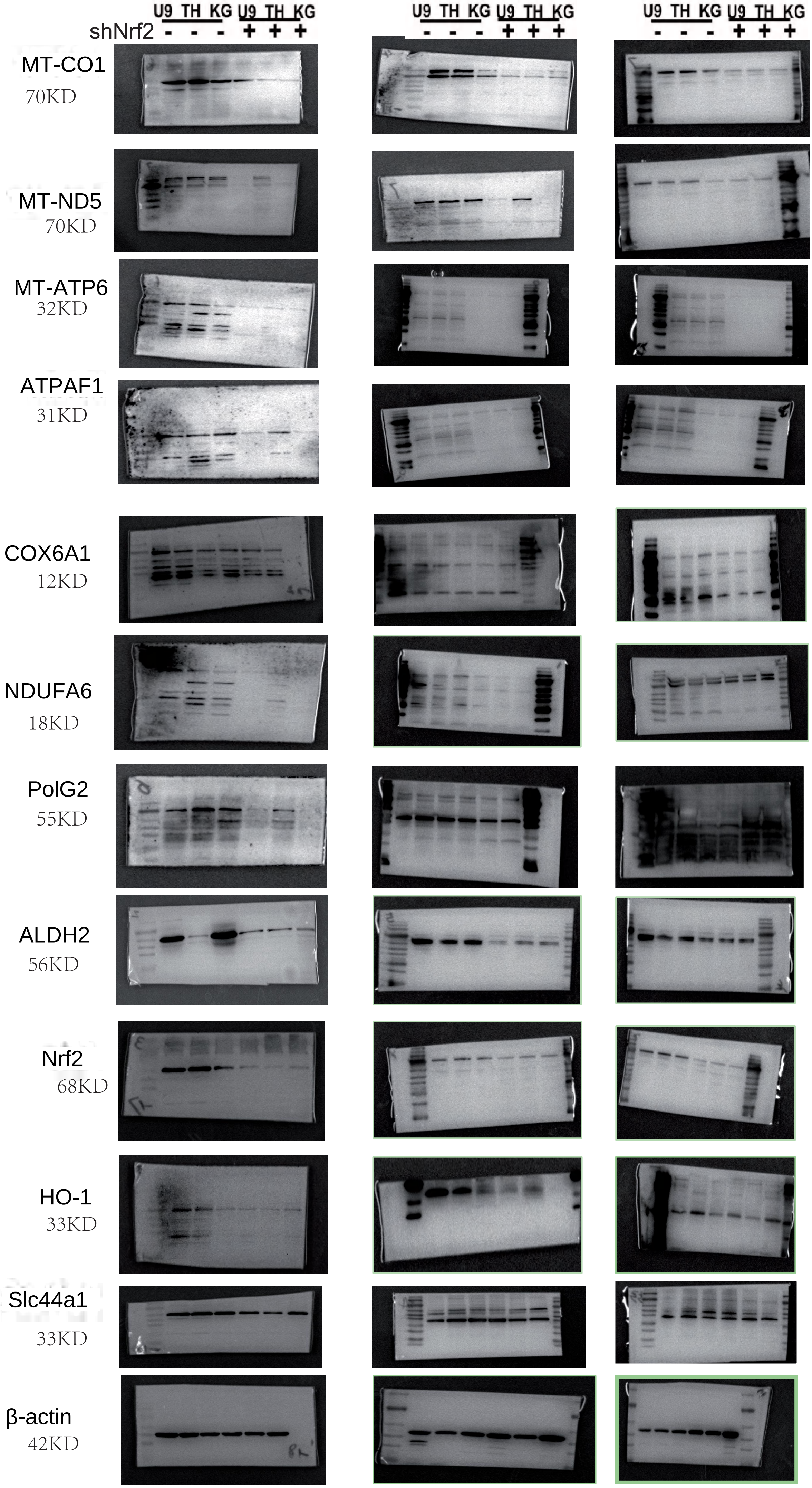

Repeat 1
Repeat 2
Repeat 3
U9 TH KG U9 TH KG
shNrf2 - - - + + +
MT-CO1
70KD
MT-ND5
70KD
MT-ATP6
32KD
ATPAF1
31KD
COX6A1
12KD
NDUFA6
18KD
PolG2
55KD
ALDH2
56KD
Nrf2
68KD
HO-1
33KD
Slc44a1
33KD
β-actin
42KD

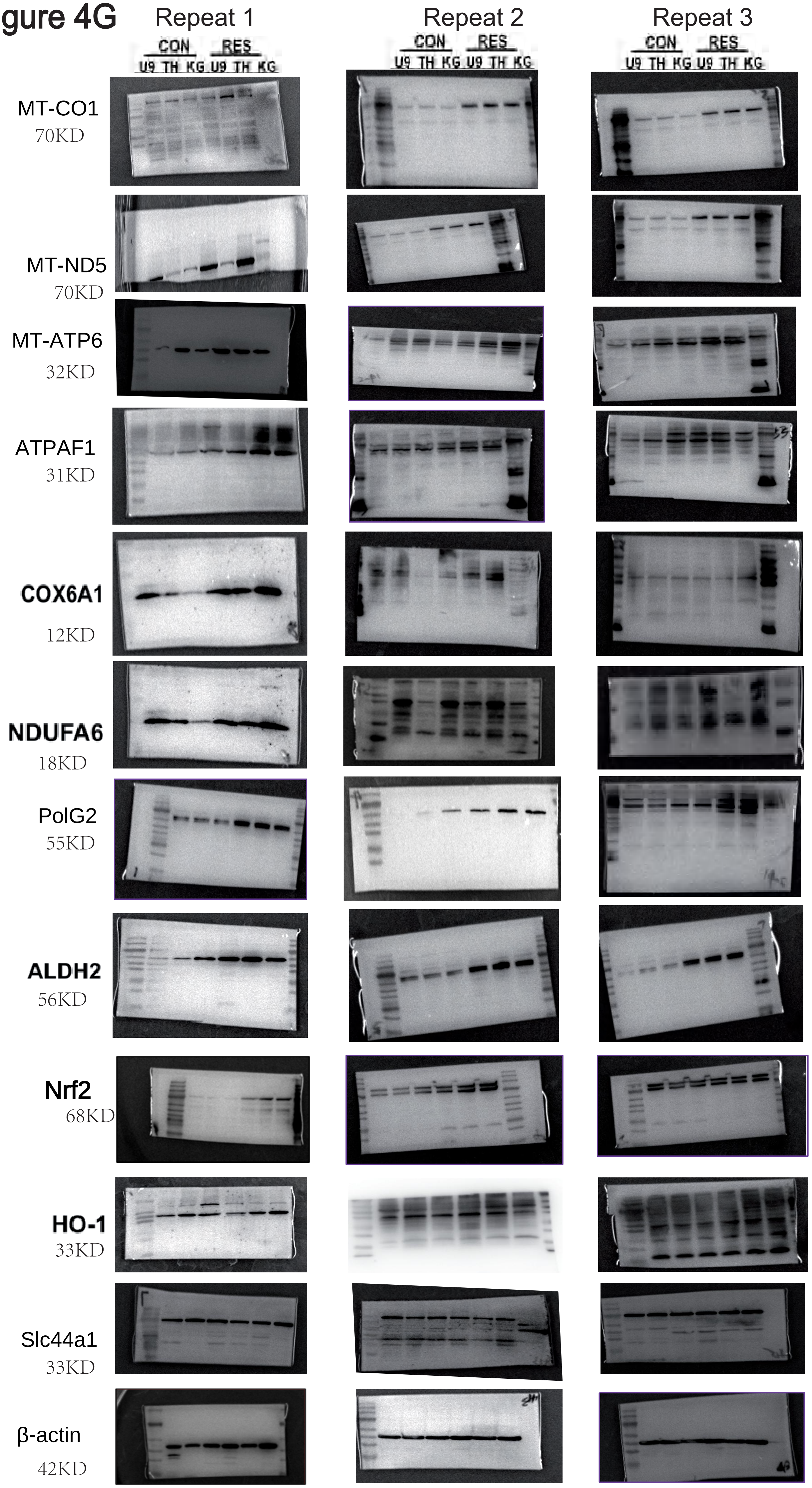

Repeat 1
Repeat 2
Repeat 3
CON
RES
U9 TH KG U9 TH KG
MT-CO1
70KD
MT-ND5
70KD
MT-ATP6
32KD
ATPAF1
31KD
COX6A1
12KD
NDUFA6
18KD
PolG2
55KD
ALDH2
56KD
Nrf2
68KD
HO-1
33KD
Slc44a1
33KD
β-actin
42KD

Figure 4G

Figure 4H

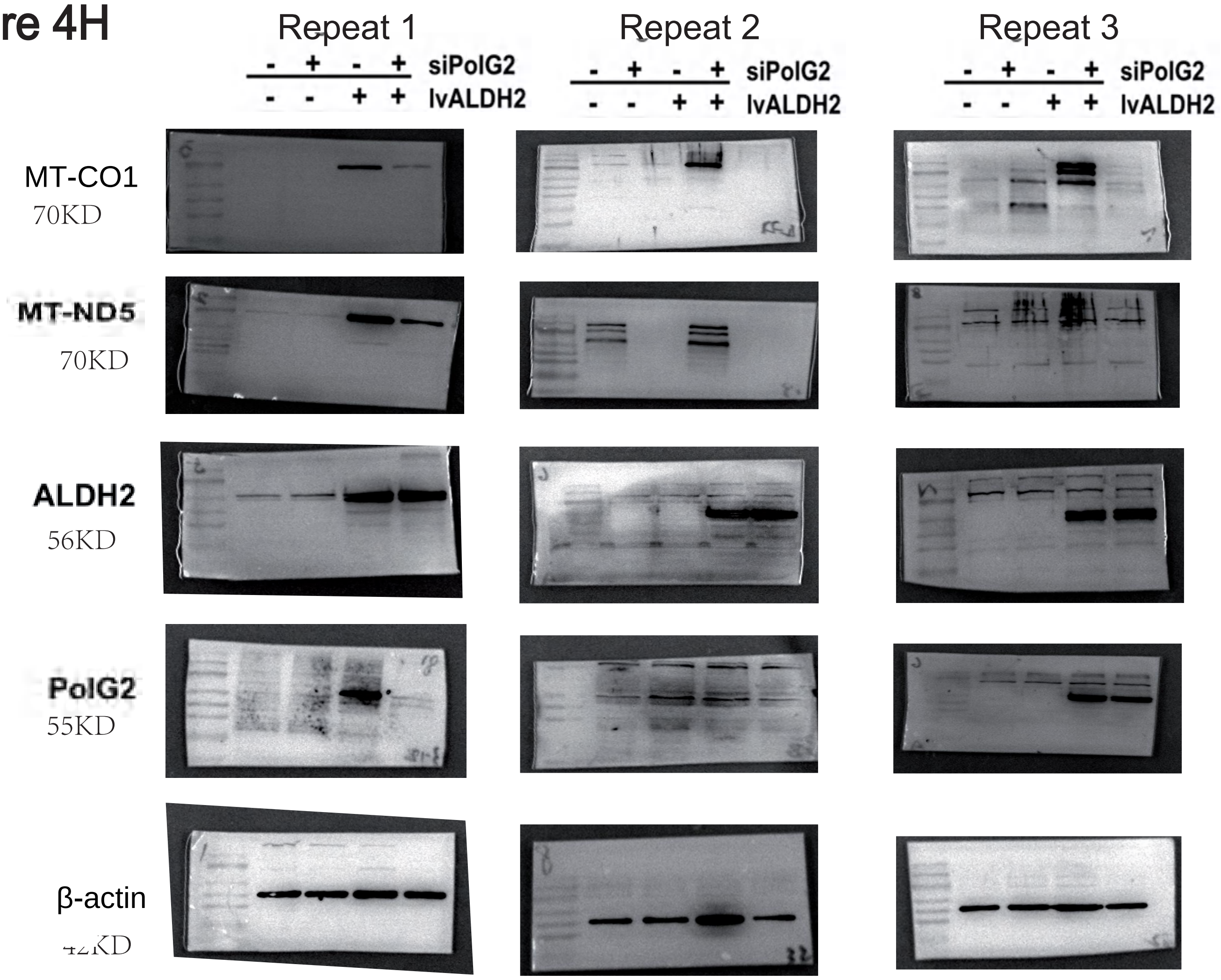
Repeat 1
Repeat 2
Repeat 3
- + - + siPolG2
- - + + lvALDH2
MT-CO1
70KD
MT-ND5
70KD
ALDH2
56KD
PolG2
55KD
β-actin

Figure 4I

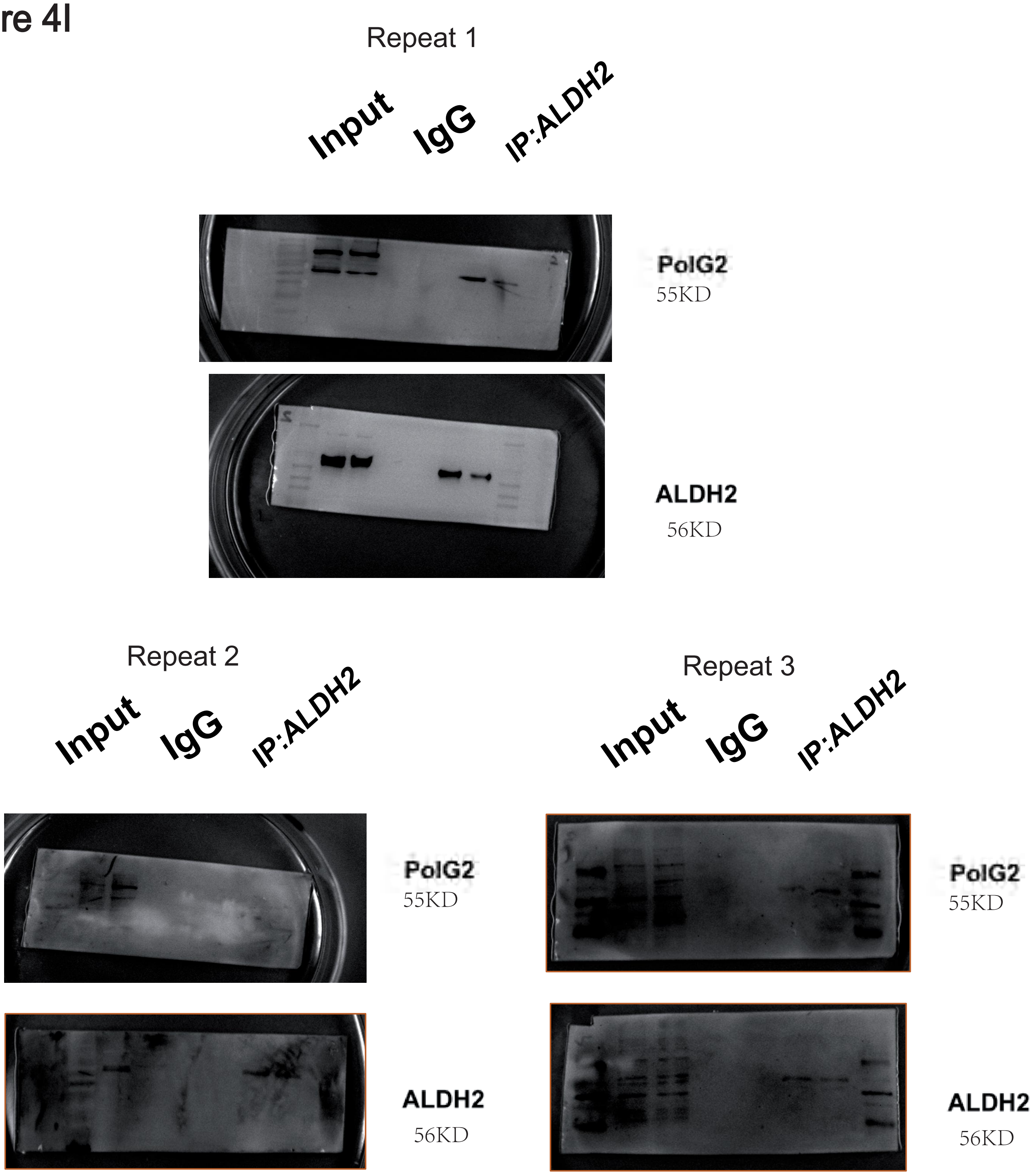
Repeat 1
Input
IgG
IP:ALDH2
PolG2
55KD
ALDH2
56KD
Repeat 2
Input
IgG
IP:ALDH2
PolG2
55KD
ALDH2
56KD
Repeat 3
Input
IgG
IP:ALDH2
PolG2
55KD
ALDH2
56KD

Figure 6K

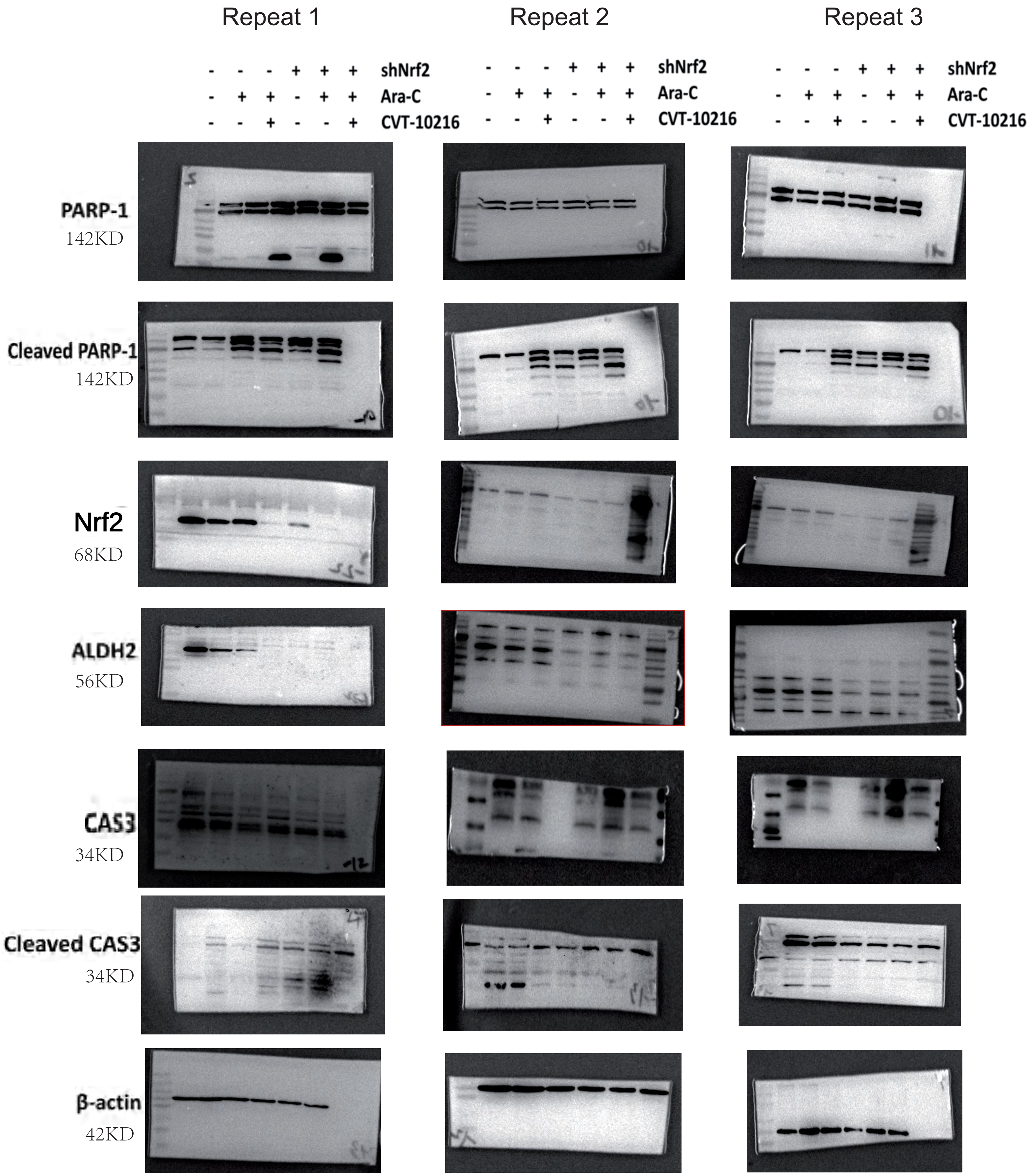
Repeat 1
Repeat 2
Repeat 3
shNrf2
Ara-C
CVT-10216
PARP-1
142KD
Cleaved PARP-1
142KD
Nrf2
68KD
ALDH2
56KD
CAS3
34KD
Cleaved CAS3
34KD
β-actin
42KD
